# A Scoping Review of Supply Chain Management Systems for Point of Care Diagnostic Services: Optimising COVID-19 Testing Capacity in Resource-Limited Settings

**DOI:** 10.3390/diagnostics11122299

**Published:** 2021-12-08

**Authors:** Kuhlula Maluleke, Alfred Musekiwa, Kabelo Kgarosi, Emily Mac Gregor, Thobeka Dlangalala, Sphamandla Nkambule, Tivani Mashamba-Thompson

**Affiliations:** 1 School of Health Systems and Public Health, Faculty of Health Sciences, University of Pretoria, Pretoria 0084, South Africa; alfred.musekiwa@up.ac.za (A.M.); thobekadlangalala@gmail.com (T.D.); 2 Department of Library Services, Faculty of Health Sciences, University of Pretoria, Pretoria 0084, South Africa; kabelo.kgarosi@up.ac.za; 3 School of Medicine, Faculty of Health Sciences, University of Pretoria, Pretoria 0084, South Africa; macgregor.m.emily@gmail.com; 4 Discipline of Public Health Medicine, School of Nursing and Public Health, University of KwaZulu-Natal, Durban 4000, South Africa; nkambulesj@gmail.com; 5 Faculty of Health Sciences, University of Pretoria, Pretoria 0084, South Africa; tivani.mashamba-thompson@up.ac.za

**Keywords:** point of care diagnostic services, supply chain management, COVID-19, resource-limited settings

## Abstract

Background: Point of care (POC) testing has enabled rapid coronavirus disease 2019 (COVID-19) diagnosis in resource-limited settings with limited laboratory infrastructure and high disease burden. However, the accessibility of the tests is not optimal in these settings. This scoping review mapped evidence on supply chain management (SCM) systems for POC diagnostic services to reveal evidence that can help guide future research and inform the improved implementation of SARS-CoV-2 POC diagnostics in resource-limited settings. Methodology: This scoping review was guided by an adapted version of the Arksey and O’Malley methodological framework. We searched the following electronic databases: Medline Ovid, Medline EBSCO, Scopus, PubMed, PsychInfo, Web of Science and EBSCOHost. We also searched grey literature in the form of dissertations/theses, conference proceedings, websites of international organisations such as the World Health Organisation and government reports. A search summary table was used to test the efficacy of the search strategy. The quality of the included studies was appraised using the mixed method appraisal tool (MMAT) version 2018. Results: We retrieved 1206 articles (databases *n* = 1192, grey literature *n* = 14). Of these, 31 articles were included following abstract and full-text screening. Fifteen were primary studies conducted in LMICs, and 16 were reviews. The following themes emerged from the included articles: availability and accessibility of POC diagnostic services; reasons for stockouts of POC diagnostic tests (procurement, storage, distribution, inventory management and quality assurance) and human resources capacity in POC diagnostic services. Of the 31 eligible articles, 15 underwent methodological quality appraisal with scores between 90% and 100%. Conclusions: Our findings revealed limited published research on SCM systems for POC diagnostic services globally. We recommend primary studies aimed at investigating the barriers and enablers of SCM systems for POC diagnostic services for highly infectious pathogens such SARS-CoV-2 in high disease-burdened settings with limited laboratory infrastructures.

## 1. Background

The primary goal of severe acute respiratory syndrome coronavirus type 2 (SARS-CoV-2) testing is to reduce the spread of coronavirus disease 2019 (COVID-19) [1,2]. Due to the highly infectious nature of SARS-CoV-2, there is an urgent need for a fast turnaround of results to institute preventative measures such as the isolation of confirmed cases and contact tracing [1,3]. Currently, reverse transcription polymerase chain reaction (RT-PCR) tests are the gold standard for diagnosing COVID-19 [4,5,6]. The laboratory equipment required to perform RT-PCR is often lacking in resource-limited settings, hindering the fast and accurate detection of SARS-CoV-2 [7,8]. 

To ease the burden on health facilities and laboratory services, alternative diagnostic methods such as point of care (POC) testing may improve the disease diagnosis [7,8]. POC testing refers to diagnostic testing that enables near-patient disease diagnosis to inform clinical decisions [9]. The benefits of POC tests are numerous, including affordability, ease of use and able to be deployed both at the site of triage and outside healthcare facilities to guide disease management [4,10]. POC tests deliver prompt results; therefore, they are of utmost importance in containing highly infectious diseases such as COVID-19 [10].

The WHO recommended scaling up testing programmes for SARS-CoV-2 by testing all suspected cases [1,10]. This recommendation was prompted by a resurgence of COVID-19 and the limited testing capacity in settings that have poor access to laboratory infrastructures [11]. The use of POC testing would significantly increase the testing capacity and allow for more accurate reporting and management of SARS-CoV-2. Rapid antigen tests also allow for the decentralisation of SARS-CoV-2 testing, thus increasing testing coverage, which may allow policymakers to institute effective adaptive policy responses [1]. To ensure the equitable availability and accessibility of POC tests, efficient supply chain management (SCM) is necessary. Supply chain refers to resources and processes needed to deliver goods and services to consumers with complete satisfaction in a cost-optimized manner [12,13]. SCM is a multifaceted system that involves production, selection, quantification, procurement, storage, distribution, redistribution, quality assurance and inventory management [10,14]. 

Evidence in supply chain systems for POC diagnostics is not clear nor readily available. This scoping review is aimed at mapping the evidence of SCM systems of all existing POC diagnostic services in order to reveal gaps to guide future research. It is also anticipated that the results of this review will help guide POC diagnostics implementers in implementing sustainable SCM for POC diagnostics to help manage highly infectious pathogens such as SARS CoV-2 in resource-limited settings. For the purposes of this study, resource-limited settings are defined as settings characterised with having limited access to laboratory infrastructures and limited capability to provide care for life-threatening illness and limited basic critical care resources.

## 2. Methodology

This scoping review was conducted as part of a multi-phase PhD study aimed at developing a novel approach for improving the SCM of SARS-CoV-2 POC diagnostic services in resource-limited settings. This scoping review protocol was registered with the Open Science Framework (OSF) under the title: “A Scoping Review Protocol for supply chain management systems for point of care diagnostics services: Optimising COVID-19 testing capacity in resource-limited settings” File S1. The published methodology was made available on 19 September 2021 for public comments via the link below: https://doi.org/10.17605/OSF.IO/RHGEF. 

This review was guided by the methodological framework proposed by Arksey and O’Malley 2005 [15] and further advanced by Levac et al., 2010 [16]. According to this framework, we conducted the review in the following five stages: (i) identify the research question; (ii) identify relevant studies; (iii) select eligible studies; (iv) charting the data and (v) collating, summarising and reporting the results. Arksey and O’Malley 2005 proposed a sixth optional stage comprising consultations with key stakeholders to provide insights beyond those found in the literature [15]. This scoping review did not include consultations with stakeholders. 

The results of the scoping review were presented according to the Preferred Reporting Items for Systematic Reviews and Meta-Analysis Extension for Scoping Reviews (PRISMA-ScR) [17]. 

### 2.1. Identification of the Research Question

The research question for this study was: What is the evidence in SCM systems for POC diagnostics services globally? To determine the eligibility of the proposed research question for a scoping review, we used the Population, Concept and Context (PCC) framework, as depicted in Table 1. 

### 2.2. Identification of Relevant Studies

We conducted a comprehensive and reproducible literature search using the following electronic databases: Medline Ovid, Medline Elton B. Stephens Company (EBSCO), Scopus, PubMed, PsychInfo, Web of Science and EBSCOHost. We also searched for grey literature, including dissertations/theses, conference proceedings, websites of international organisations such as WHO and government reports. We identified additional relevant studies by manually searching all references cited in the included studies to identify studies not indexed in electronic databases. Language restrictions were not applied to minimise the risk of excluding relevant studies.

This comprehensive search strategy was codeveloped by the principal investigator (PI), subject specialist and university librarian to ensure the correct use of indexing terminology and Medical Subject Headings (MeSH) terms. The following keywords or MeSH terms were used: (1) “supply chain management” or “supply chain” or “supply chain flow” or “supply chain systems”, (2) “point of care” or “point of care testing” or “point of care diagnosis” or “point of care diagnostic services” and (3) “SARS-CoV-2” or “COVID-19” or “Coronavirus”. The keywords were refined to suit each database. Each search was documented in detail, showing the keywords/MeSH terms, date of search, electronic database and number of retrieved studies, and the results of the search were tabulated in File S2.

The search strategy was optimised by adopting the search summary table (SST) outlined by Bethel et al. [19] as a guide. The SST was used to improve and report on the effectiveness of the search strategy. 

### 2.3. Selection of Eligible Articles 

This scoping review was guided by inclusion and exclusion criteria to ensure the correct identification and selection of relevant articles. 

#### 2.3.1. Inclusion Criteria

The included articles met the following criteria:

- Articles reporting evidence on SCM systems of all diseases
- Articles reporting evidence of SCM systems for all POC diagnostics services at all levels of the healthcare continuum
- Articles reporting evidence of primary studies conducted in LMICs
- All reviews providing evidence of SCM systems for all POC diagnostic services
- Articles published since inception

#### 2.3.2. Exclusion Criteria 

Articles were excluded from the scoping review if they had the following characteristics:

- Articles that lacked evidence on SCM systems for all POC diagnostics services
- Articles reporting SCM systems of laboratory-based POC diagnoses
- Articles reporting evidence of primary studies conducted in high-income countries

### 2.4. Selection of Sources of Evidence 

The articles were screened in three stages, namely title, abstract and full-article screening. Reviewers used a screening tool (File S3) developed by the PI and pilot tested by the reviewers. The eligible articles were exported to an Endnote 20 library, and the duplicates were removed. The PI screened abstracts in parallel with the co-reviewer (EM). After screening the abstracts, the reviewers discussed any discrepancies in the selected articles until a consensus was reached. Two reviewers (KM and EM) then screened the full texts of articles selected during the first stage. A third screener (TD) resolved any discrepancies in the selected articles after full-text screening. Both abstract and full-article screening were guided by the screening tool that factored all aspects of the inclusion/exclusion criteria and the PCC elements. 

The level of agreement between screeners’ results after screening the abstracts and full articles was determined by calculating Cohen’s kappa statistics. The kappa statistics were interpreted as follows: values <0.1 indicate no agreement and 0.10–0.20 indicate none to slight, 0.21–0.40 as fair, 0.41–0.60 as moderate, 0.61–0.80 as substantial and 0.81–1.00 as almost perfect agreement.

### 2.5. Charting the Data

Data were captured from each included article using a data charting form. Two independent reviewers (KM and TD) piloted the data charting form and recommended modifications that were implemented. The following data were extracted from the included articles: author and year of publication, title of study, aim of study, country, study design, study setting, study population, type of point-of-care test investigated, stage of SCM investigated, main findings and other significant findings.

### 2.6. Collating, Summarizing and Reporting the Results

We thematically analysed the data extracted from the included articles. The themes were narratively summarised. The following themes emerged from the data: availability and accessibility of POC diagnostic services; reasons for stockouts of POC diagnostic tests (procurement, storage, distribution, inventory management and quality assurance) and human resources capacity in POC diagnostic services.

### 2.7. Quality Appraisal

We used the mixed method appraisal tool (MMAT) version 2018 to evaluate the quality of the included articles [20]. Using MMAT, we appraised the methodological quality of five categories of research: qualitative research, randomised controlled trials, nonrandomised studies, quantitative descriptive studies and mixed methods studies [20]. Two independent reviewers (KM and TD) carried out the quality appraisal process. The following percentage scores were used to grade the quality of evidence: (i) ≤50% represented low-quality evidence, (ii) 51–75% represented average-quality evidence and (iii) 76–100% represented high-quality evidence.

### 2.8. Ethical Considerations

This scoping review relied on a synthesis of the existing literature, and therefore, ethical approval was not required.

## 3. Results

### 3.1. Screening Results

**Figure 1:**
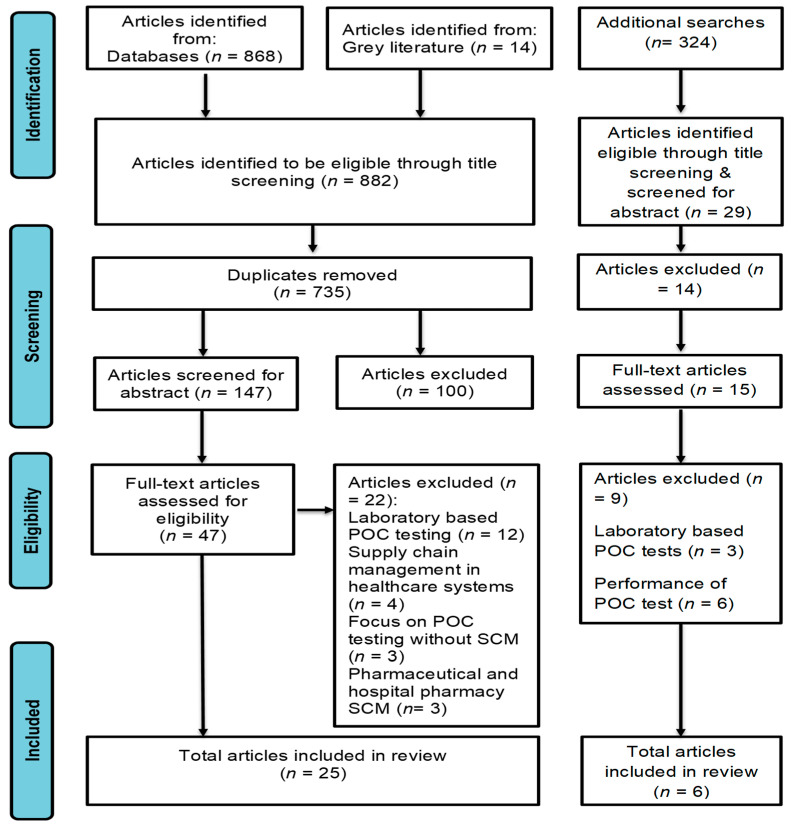
PRISMA-ScR flow chart showing the literature search and selection of articles.

Our screening search involved title screening and returned a total of 882 results, which consisted of 868 articles and 14 articles from grey literature (Figure 1). Following the removal of 735 duplicate articles, 147 remained. The 147 articles were included in abstract screening. During abstract screening, 47 articles met the inclusion criteria for full-article screening, and 100 were excluded. Of these 47 articles that were included in full-article screening, 22 did not meet the inclusion criteria, and they were excluded. The data were extracted from the remaining 25 articles. The reasons for excluding the 22 articles included were as follows: 12 articles focused on laboratory-based POC diagnostic services, 3 articles did not discuss any aspect of the SCM system, 4 articles focused on the SCM of health systems and 3 articles focused on pharmaceutical and hospital pharmacy SCM. An additional search was performed on the 28th of October 2021, and it returned 324 articles. Based on the title screening, 29 articles were found eligible and screened for abstracts. After abstract screening, we excluded 14 articles, and 15 articles met the inclusion criteria for full-article screening. Of these, we excluded 9 articles. Additional data were extracted from the remaining six articles. The reasons for excluding the nine articles included: three articles focused on laboratory-based POC tests, and six articles focused on evaluating the performances of the POC tests. After full-text screening, there was a high agreement of 96.77% vs. 50% expected by chance (Kappa statistics = 0.93, *p* < 0.05). In addition, McNamar’s chi-square statistics suggested no significant differences in the proportions of yes/no answers by the reviewers (*p* > 0.05) (File S4). The search strategy was continuously improved. An updated search (rerun) was essential, because SARS-CoV-2 is a novel virus, and new research is published frequently (File S5).

### 3.2. Characteristics of the Included Articles

**Figure 2:**
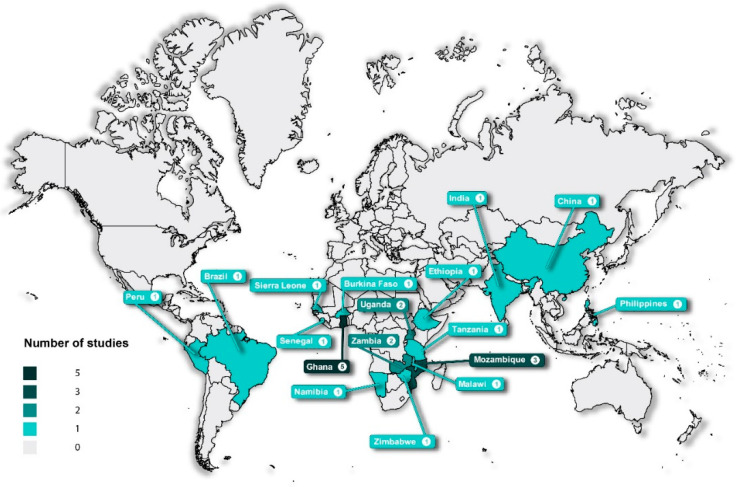
World map showing the distribution of the articles included in the scoping review on supply chain management systems for point-of-care diagnostic services.

The characteristics of the included articles are summarised in Table 2. The eligible studies were published between 2011 and 2021. The 31 articles included 15 reviews [10,12,21,22,23,24,25,26,27,28,29,30,31,32], 1 website of an international organisation [33], 2 randomised controlled trials [34,35], 1 cross-sectional study [36], 1 case study [37], 1 cohort study [38] and 10 studies that used mixed method approaches ranging from focus group discussions to interviews to direct observations [39,40,41,42,43,44,45,46,47,48]. The included articles presented evidence of research conducted in the following countries: Mozambique [34,36,44], Zimbabwe [49], Ghana [12,21,22,40,45,48], Malawi [42], Namibia [41], India [43], Sierra Leone [37], Burkina Faso [38], The Philippines [38], Senegal [38,47], Ethiopia [38], Uganda [39,46], Brazil [39], Peru [39], China [39], Tanzania [39] and Zambia [35,39]. Figure 2 shows the distribution of eligible articles by country. Peru, Brazil, China, Namibia, The Philippines and India are middle-income countries, and Mozambique, Ghana, Malawi, Sierra Leone, Burkina Faso, Senegal, Ethiopia, Uganda, Tanzania, Zimbabwe and Zambia are low-income countries. Two articles were conducted in more than one study setting. A cohort study by Albertini et al. was conducted in Senegal, Burkina Faso, Ethiopia and The Philippines [38]. A mixed methods qualitative study by Mabey et al. was conducted in Peru, Brazil, China, Uganda, Zambia and Tanzania [39].

**Figure 3:**
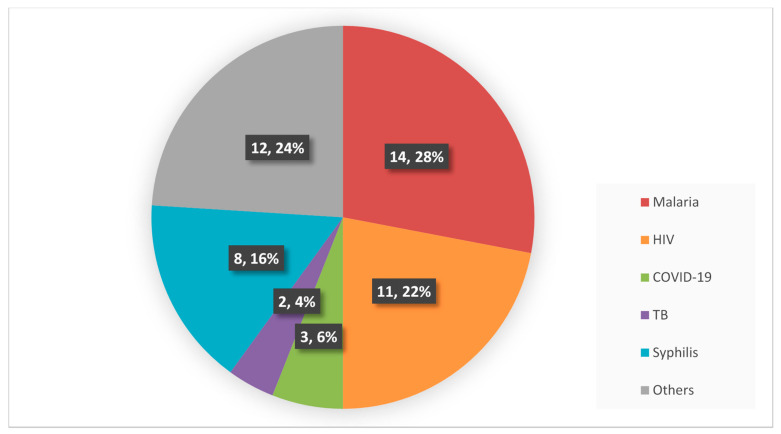
Distribution of the types of POC diagnostic tests in the articles included in the scoping review of supply chain management systems for point-of-care diagnostic services.

Evidence on the following POC diagnostic tests were provided: COVID-19 [27,28,33]; HIV [10,12,22,23,24,25,26,34,36,42,49]; malaria [10,12,22,23,34,35,38,40,41,43,44,46,47,48]; syphilis [10,12,23,32,34,39,42,45]; tuberculosis [30,31] and others comprising of anaemia [34,40], UTI [37], diabetes [12,31], blood glucose [22], preeclampsia urinalysis [37], urine pregnancy [22], urine protein [22,34], haemoglobin [22], dyslipidaemia [12], dengue [30], meningitis [29], hepatitis C [29] and hepatitis B [12,22,29,30,31]. Figure 3 shows the types of POC diagnostic tests in the included articles. Malaria POC diagnostic tests were investigated in 14 articles, HIV POC diagnostic tests in 11 articles, syphilis POC diagnostic tests in 8 articles, COVID-19 POC tests in 3 articles, TB POC tests in 2 articles and the other POC diagnostic tests were investigated in 12 articles.

**Figure 4:**
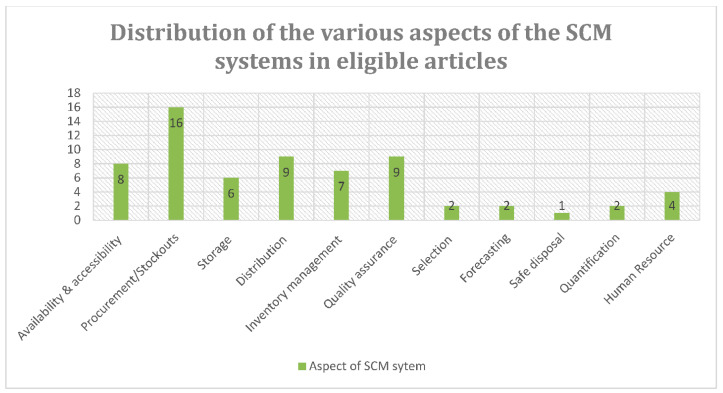
Aspects of supply chain management systems in the articles included in the scoping review on supply chain management systems for point-of-care diagnostic services.

The eligible studies provided evidence on at least one aspect of the SCM system. The aspects of the SCM systems covered were procurement and stockouts [10,12,21,24,25,26,33,34,36,41,42,43,44,45,47,49], quality assurance [10,23,24,27,29,31,32,37,46,48,49], safe disposal [46], inventory management [10,12,21,25,35,36], selection [10,22,28,33,46], distribution [12,21,22,34,38,41,48], quantification [12], storage [12,21,38,41,48], human resource capacity [30,35,40,47] and the availability and accessibility of POC diagnostic services [10,12,23,32,39,47,48]. Figure 4 shows the aspects of the SCM systems described in the included articles.

### 3.3. Quality of Evidence

From the 31 included articles, 15 primary studies were appraised for methodological quality using MMAT version 2018 [20]. The remaining 10 articles were excluded because they were not primary studies [10,12,21,22,23,24,25,26,32,49]. After categorising each study, we rated the criteria of the chosen categories to establish whether the criteria for each study design had been met. The 15 studies that were appraised for methodological quality scored between 90% and 100%, which showed high methodological quality. Twelve studies scored the highest quality score of 100% [34,35,36,39,40,41,42,43,44,45,47,48]. One study scored 95% [38]. The remaining two studies scored 90% and 92% [37,46] (File S6).

### 3.4. Main Findings

All the included articles presented evidence of at least one aspect of SCM of POC diagnostic services globally. The following themes emerged from the included articles: availability and accessibility of POC diagnostic services, reasons for stockouts of POC diagnostic tests (procurement, storage, distribution, inventory management and quality assurance) and human resources capacity in POC diagnostic services.

#### 3.4.1. Accessibility and Availability of POC Diagnostics Services

Eight articles reported evidence on the availability and accessibility of POC diagnostic services [10,12,23,27,34,39,47,48]. These articles showed that the accessibility and availability of POC diagnostic services in resource-limited PHC clinics contributed to improved health outcomes [12]. A review conducted by Peeling [23] showed that, in Peru, it would take five to six visits spread over 27 days to screen and treat pregnant women for syphilis. With the introduction of rapid diagnostic tests (RDTs), the testing and treatment of infected mothers was done on the same day [23]. A randomised controlled trial conducted in Mozambique found that introducing POC diagnostic testing for pregnant women improved antenatal care [34]. A significant improvement in the accessibility of antenatal POC diagnostic tests was reported in all implementation sites. In the first visits, 5519 (14.6%) out of 37,826 women were screened for anaemia compared to 30,057 (97.7%) out of 30,772 in the intervention, 3739 (9.9%) out of 37,826 women were screened for proteinuria during the control period compared with 29,874 (97.1%) out of 30,772 in the intervention period and 17,926 (51.4%) out of 34,842 received mebendazole during the control period compared with 24,960 (88.2%) out of 28,294 during the intervention period [34].

In a survey conducted by Poole et al., 2021 highlighting the current state of RDTs globally, most of the respondents reported 24/7 availability of tests [29]. High-income countries reported a higher availability of the rapid influenza test compared to low-income countries [29]. Low-income countries reported a high availability of HIV and hepatitis rapid tests. The reason given for this pattern is that hepatitis and HIV are more prevalent in developing countries, where public health interventions are less likely to identify and treat patients early during illness [29]. Another reason is that the priorities for treatment are different: influenza management in secondary care is a less-pressing need in resource-limited settings where patient isolation facilities are less readily available [29]. Additionally, the cost of identifying an influenza case is less than HIV or hepatitis, where early identification and treatment make a greater difference. The cost of each test is likely to also be a factor in the difference of availability, with multiplexed assays generally being considerably more expensive and requiring more complex logistical support. Methods for reducing the costs of many RDTs are lacking, which limits their availability in low-income settings [29].

Decentralisation was an important factor promoting the accessibility and availability of POC diagnostic services. The decentralisation of POC diagnostic services increased detection, patient management and prompt treatment initiation when necessary [23,32,39]. Mabey et al., 2012 reported on the introduction of POC testing for syphilis in various urban (China and Peru) and rural settings (villages in East Africa). The proportion of antenatal care attendees increased by more 90% in areas that previously had some testing [39]. In areas where syphilis POC diagnostic testing had previously not been available, the screening rate increased by more than 50% [39]. In all settings, more than 90% of those who tested positive received syphilis treatment [39].

In most of the studies, POC diagnostic services relied solely on government supplies [35,37,39,41,42,43,45]. Two articles reported that the supply of POC diagnostic tests was insufficient and sporadic [12,48]. This affected malaria testing in a study conducted in Ghana, which determined the factors that influenced the implementation of POC diagnostic tests in routine malaria management at PHC facilities [48]. At the time of the study, four study sites had limited malaria RDTs, while two sites had none [48]. As RDTs became less available, the rate of blind treatment increased. Other patients opted to buy test kits privately, causing financial strain [48]. The RDT supplies were interrupted due to frequent and prolonged RDT stockouts [48].

#### 3.4.2. Reasons for Stockouts of POC Diagnostic Tests

The reviewed articles revealed that stockouts of POC diagnostic tests were caused by problems in procurement, inventory management, storage, distribution and quality assurance.

- Procurement of POC diagnostic tests

Sixteen articles reported stockouts due to procurement issues [10,12,21,24,25,26,34,36,40,41,42,43,44,45,47,49]. Of the 16 articles, one article reported an adequate inventory of test kits more than 89% of the time across the 75 facilities from a study conducted in Mozambique [36]. In this study, the HIV RDT stock levels were well-maintained due to technical support received from a nongovernment organisation affiliated with the Global Fund [36]. The stock levels were also monitored during monthly visits and followed up with planning and coordinating supply chain logistics with health facilities [36].

The other 15 articles reported poor procurement processes. Two qualitative studies conducted by Magesa et al., 2019 and Maddox, et al., 2017 reported that more than 60% of the key informants from all levels of the supply chain reported stockouts due to a lack of proper knowledge and training in procurement processes [41,42]. A randomised control trial conducted by Betrán et al. [34] reported that, during the intervention period, two clinics had a single 3-day period with no kits due to clinics making late requisitions to the health directorate [34]. According to Hussain, Dandona, David and Schellenberg [43], malaria diagnoses were compromised by a failure of the supply chain due to poor communication or procurement systems compromising the availability of POC tests at PHC facilities. Almost half (48%) of the facilities did not have test kits. Similar findings were reported by Hasselback et al. [44], where 59% of health facilities reported stockouts due to poor communication between health facilities and suppliers [44]. This resulted in a high rate of referral to private healthcare facilities, which hindered the early diagnosis and complete treatment of malaria [43].

The WHO, in a document that aims to bring clarity on the process of requesting and receiving globally sourced COVID-19 critical diagnostics supplies, stated that low- and middle-income countries have continued to experience restrictions in test access due to competition for limited volumes with high-income countries [33]. Manufacturers have also faced challenges in scaling up manufacturing to meet all testing needs. Prices for diagnostic products remain high, and some national governments continue to face restricted access to tests. To access POC tests, purchasers may place orders directly with the companies or utilize one of the available multilateral procurement channels. The funded demand and requests are being followed closely to determine whether these tests may be constrained in volume availability. If they become constrained, an allocation model using the same principles as above will be implemented to ensure equitable distribution [33].

- Inventory management of POC diagnostic tests

Consumption data was important to guide the quantity of inventories procured at all implementation sites [47]. Adequate inventory was promoted by a well-managed and monitored system to enable clear communication between PHC facilities and regional or provincial offices [24,41,45]. Lack of consumption data contributed to stockouts of malaria RDTs as a results of a poorly functioning Facility Electronic Stock Card (FESC) in Namibian PHC facilities [41]. A lack of consumption data resulted in a high rate of expired medicine and wastage of malaria RDTs due to overstock, while other study sites experienced stockouts due to underestimated consumption [41]. In their review, Peeling et al., 2015 concluded that real-time data monitoring via electronic readers improved inventory management [23]. Operational data on stocks, device usage and conditions were uploaded via Wi-Fi or cellular networks and transmitted to central databases [23]. Linking the data to SCM software helped avoid stockouts and improved the efficiency of the health system [23].

In Mozambique, 15 health facilities were surveyed over 120 time points [44]. Stockout patterns varied by data source, with an average of 59% of health centres reporting stockouts on stock cards every month, preventing the proper documentation of consumption data [44]. Each ten-unit increase in monthly-observed consumption was associated with a nine-unit increase in lost consumption, indicating higher rates of stockouts at higher levels of observed consumption [44]. Stockouts were caused by the inaccurate tracking of lost consumption, insufficient sophistication in inventory management and replenishment and poor process compliance by facility workers, all stemming from inadequate attention to design and implementation of the inventory management system [44].

- Storage of POC diagnostic tests

Six articles mentioned that RDTs should be stored under recommended temperatures, because exposure to adverse environmental conditions had the potential to degrade POC diagnostic tests [12,21,37,38,41,48]. A study investigating storage and temperature conditions in Burkina Faso, Senegal and Ethiopia revealed that malaria RDTs were being stored at temperatures exceeding the recommended RDT manufacturer temperature limit [38]. In three of the eight facilities in Burkina Faso, temperatures rose above the recommended RDT manufacturer temperature limit of 40 °C [38]. In 11 of the 13 facilities in Ethiopia, temperatures exceeded the recommended RDT manufacturer temperature limit of 30 °C. In five out of ten facilities in Senegal, temperatures exceeded the recommended RDT manufactured limit of 40 °C [38]. In this study, RDTs were exposed to unfavourable conditions for only brief periods, making it difficult to detect if product shelf lives were shortened [38]. When RDTs are exposed to extreme conditions for some time, it is costly to retest a withdrawn batch, and the continuity of diagnostic services is disrupted [38,46]. Ways to lower temperatures were explored in Uganda [46]. This was done by implementing techniques such as underground storage and the use of evaporative cooler boxes. These studies concluded that RDTs should be selected according to the expected field conditions [38,46].

An association between the limited storage capacity contributed and stockouts was reported in Namibia [41]. Most clinical settings (*n* = 16, 94%) reported problems with storage resulting in the expiration of diagnostic tests. Stock was stored in the wrong place, and staff were not always aware that there was stock. Some study sites would pile up boxes on the floor, compromising the qualities of the diagnostic tests [41].

Evidence has reported the robustness of syphilis rapid tests, because they do not require special storage or transport conditions [32]. Syphilis tests can be stored at temperatures ranging from 8 to 30 °C [32]. If clinics are warmer than 30 °C, the shelf life of rapid tests is reduced, and sensitivity is lost [32]. In settings that rely on POC diagnostic tests, it is important to conduct periodic quality control checks to ensure ongoing validity. Health authorities should visit study sites to ensure that POC diagnostic tests are functioning as expected [32].

In Ghana, malaria RDTs are not always distributed timeously, and there is limited storage space at the district office, resulting in RDTs being left outdoors, exposed to sunlight and other weather conditions [48]. Providers were concerned with compromised test qualities due to poor storage, and they had little confidence in the accuracy of the test results [48].

- Distribution of POC diagnostic tests

Nine articles discussed the distribution of POC diagnostic tests and highlighted that regional or national health directorates were responsible for distributing POC diagnostic tests to PHC clinics [12,21,22,34,38,41,48]. The timely distribution of POC diagnostic tests to PHC clinics often depends on the availability of tests at the regional or national health directorate. In Ghana, Kuupiel, Tlou, Bawontuo, Drain and Mashamba-Thompson [22] found that 90% of the PHC clinics received POC diagnostic supplies within 24 h after requisition. While the distribution of POC tests was adequately managed, PHC staff revealed documentation challenges that would limit their ability to forecast demands. They further recommended that PHC staff require training on the documentation of test stock levels to aid forecasting demands to ensure the continued supply of diagnostic tests to match consumption [22].

In Ghana, healthcare providers reported their perspectives and experiences in the rollout of rapid POC tests for antenatal syphilis screening [45]. Almost half (6/13) of the facilities that had started antenatal syphilis screening did not have any syphilis test kits, while HIV test kits were available in all the 14 facilities that were screening pregnant women for HIV [45]. In some facilities, stockouts of syphilis test kits were quite frequent, but pregnant women were not referred to other facilities for testing and were only screened when the test kits became available [45]. In one region, healthcare providers blamed stockouts of test kits on inadequate regional supplies, while the regional staff blamed stockouts on the lack of returns from districts and healthcare facilities and, less commonly, insufficient supplies from national headquarters [45].

Mozambique experiences rainy seasons, which are associated with a high prevalence of malaria and distribution difficulties. Rural areas are not easily accessed due to poor road infrastructure [44]. Large trucks cannot drive on wet dirt roads sometimes, causing motorcycles to be the primary mode of distribution, which does not allow for the same protection as trucks [37]. The use of malaria RDTs increases by more than 300% in the rainy season; therefore, it is important to plan the distribution aspects to prevent stockouts during such a critical season [44].

- Quality assurance of POC diagnostic tests

Eight articles discussed the importance of following quality assurance processes when handling POC diagnostic tests [10,23,24,32,37,46,48,49]. These articles showed that poor regulatory mechanisms further limited RDT implementation. Quality assurance processes were seldom in place, preventing the ongoing monitoring of POC diagnostic tests. Infrequent quality assurance and control visits to facilities by authorities further undermined providers’ willingness to use RDTs [10,23,24,32,37,46,48,49].

Evidence revealed that the current setup of RDTs appears to be more laboratory-centred. Governance and quality control were reported to be the responsibility of laboratories in the vast majority of those surveyed [29]. A total of 90% of those who responded to the survey reported that tests were carried out in their institution by laboratory staff [29]. Simpler tests, RDTs, were conducted more during point-of-care. While there are several existing international regulatory processes for drugs and medications, providing safeguards for their safety and efficacy, they are often lacking for RDTs [29]. As a result, diagnostic tests are often sold and used in the developing world without any evidence of effectiveness. For example, a study conducted to evaluate the performance of RDTs by Mak et al. [26] reported the sensitivity of an RDT for SARS-CoV-2 of 11.1–45.7% when the manufacturer had claimed it was 98%. This is indicative of poor-quality assurance mechanisms in the sites surveyed.

#### 3.4.3. Human Resource Capacity in POC Diagnostic Services

Four articles discussed the importance of human capacity in POC diagnostic services [35,37,40,47]. Two studies reviewed how the services of community healthcare workers (CHWs) are used to assisting in providing POC diagnostic services in resource-limited settings. In Sierra Leone, Ekambaram, Gomanie and Mehta [37] reported that CHWs had no formal training and that human error contributed to losing POC tests [37]. In Senegal, Blanas et al. [47] reported that most lay health workers acquired important skills, but few did not understand the RDT algorithm soon after the training. Although the scores improved by 10–20% after two months of field training, half of the CHWs still could not interpret the RDT algorithm correctly, and almost half could not prescribe artemisinin-based combination therapy (ACT) correctly [47].

Hamer et al. [35] evaluated the ability of CHWs to provide high-quality, safe, integrated care for malaria and pneumonia in two rural districts in Zambia. Community health workers were able to manage malaria using RDTs at the community level. The CHWs performed RDTs with 90% accuracy and with 93% correctness after a 3-h training session assisted by visual job aids and RDT package inserts. With enough training, CHWs were able to handle RDTs without significant risks to themselves and their patients, reducing wastage linked to the improper use of RDTs [35]. In Zambia, CHWs also contributed to successful SCM by keeping accurate records and engaging with supervisors to ensure adequate supplies [35].

A review conducted by Kumar et al. [30] further emphasised the importance of human resource capacity in the improvement of diagnostic accuracy and precision. They reported that appropriate sample handling approaches are important in reducing errors during sampling, testing and interpretation of the POC test. For an infectious disease diagnosis, the sample could take different forms, such as urine, serum, blood, plasma, stool or saliva. The different physical properties and chemical compositions of these samples demand proper and appropriate approaches that can accommodate the target analyte in an acceptable form [30]. The Lancet commission also found that the reliability of the diagnostic test depends not just on the performance characteristics of the actual test but on all the elements involved in the testing—for example, sample collection and preparation (PALM), result interpretation and result communication [31]. All these elements rely on users being adequately trained and having ongoing access to quality control materials and technical support and maintenance for instruments. It is therefore pivotal to incorporate adequate quality assurance and quality control into the point-of-care testing protocols. Additionally, these point-of-care diagnostics should only be used in situations in which there are referral pathways and there is healthcare provider buy-in and patient trust [31].

## 4. Discussion

We conducted a scoping review to map the evidence on SCM systems for POC diagnostic services with the goal of optimising the COVID-19 testing capacity in resource-limited settings. Our scoping review results show that there is limited published research on SCM systems of POC diagnostic services. Studies have been conducted in sixteen low- and middle-income countries. This is a major public health concern and requires immediate action from all relevant stakeholders, especially since 47% of the global population has little to no access to diagnostic services [31]. The COVID-19 pandemic has emphasised the crucial role of diagnostics in healthcare and that, without access to diagnostics, the delivery of universal health coverage, antimicrobial resistance mitigation and pandemic preparedness cannot be achieved, thereby hampering the progress towards achieving sustainable development goal (SDG) 3 that aims to promote good health and well-being for all by 2030 [50]. Our scoping review findings also demonstrate that, for the continuum of POC diagnostic services, POC diagnostic tests must be available and accessible to all who need them through sustainable SCM systems [34,43,47,48]. Sustainable SCM systems are influenced by several factors, some of which are procurement, inventory management, storage, distribution, quality assurance and the human resource capacity, as revealed in most of the study settings explored [34,35,36,38,39,41,46,47]. Weak SCM systems may lead to significant stockouts of POC diagnostic tests [21,36,43]. Stockouts result in the reduced use of POC diagnostic tests in resource-limited settings, which negatively impacts health outcomes [35,39,45].

The WHO has been the main driver in ensuring that POC diagnostic tests are available in resource-limited settings through their prequalification program and bulk procurement systems [14,32]. The prequalification program facilitates access to medicines and medical equipment that meets unified standards of quality, safety and efficacy for HIV/AIDS, malaria and tuberculosis, with the aim of reducing widespread disease in countries with limited access [14,32]. Currently, the Foundation for Innovative New Diagnostics (FIND), in collaboration with WHO and other organisations, is playing a pivotal role in scaling up the development and delivery of COVID-19 tests through its Access to COVID-19 Tools (ACT) Accelerator and provide sustainable solutions beyond the COVID-19 pandemic [51]. To provide sustainable solutions in providing POC diagnostic tests and to avoid past uncoordinated procurement issues, various stakeholders have joined forces to speed up the end of the pandemic by supporting the development and equitable distribution of the tests, treatments and vaccines the world needs to reduce mortality and severe disease, restoring full societal and economic activity globally in the near term, and facilitating a high-level control of COVID-19 in the medium term [51]. The collaboration supports the coordinated, uninterrupted provision of timely, high-quality diagnostic tests in resource-limited settings [26]. The collaboration serves as a platform for information exchange, as well as alignment, on procurement principles, planning and addressing key SCM issues [26]. They also ensure that there are adequate funds to purchase projected POC diagnostic tests to make them available for use at the POC in a timeous manner.

Effective SCM is dependent on the skilled human resource capacity for POC diagnostic services [21]. Staff in PHCs should be trained in various aspects of POC diagnostic test SCM [21]. Staff should also be trained in how to perform POC diagnostic testing accurately to prevent wastage [21]. Training is also important to aid in stock management and the safe disposal of used test kits to ensure personnel and environmental safety [21]. Training programs complemented with strengthened monitoring and supervision support at the clinics may ensure compliance with SCM guidelines, and acceptable standards will further enable the suitability of diagnostic services.

Storage, distribution and inventory management are equally important in the SCM system. Regional or provincial warehouses should have enough stock to ensure adequate distribution to all PHC facilities. Regional warehouses and PHC storage facilities need to meet storage requirements. While distributing POC diagnostic tests, harsh environmental conditions should be avoided by using refrigerators and cold chain facilities [21]. Improved inventory management will ensure that enough tests are available to meet demand. Health facilities should have set minimum and maximum levels and follow the First Expired, First Out principle, thus preventing tests from expiring. Inventory management linking national, regional, district and PHC facilities could potentially benefit from the adoption of digital technology, such as blockchain. Blockchain is an emerging digital technology that has unique characteristics, such as immutability, decentralisation and transparency, that can be used for the coordination of large-scale operations such as population-level mass screening, rapid contact tracing and supply chain management [52]. This modern technology is widely used in high-income countries [53]. In resource-limited settings, the adoption of blockchain digital technology may be costly; therefore, low-cost blockchain may be more sustainable [54].

The adoption of real-time technology such as the Stock Visibility System (SVS) can bring visibility to the supply chain process, make it seamless, and can facilitate effective and efficient inventory management at all levels [55]. The SVS facilitates the detection of high- and low-consumption PHC clinics to enable the redistribution of tests to prevent the overstocking and expiration of POC tests [55]. The distribution of SARS-CoV-2 POC diagnostic tests can be optimised by using unmanned aerial vehicles (UAVs) or drones that can be operated either autonomously or remotely by humans [56]. The use of drones has been extensively adopted for the faster and safe transfer of essential products, assisting authorities to deal with and possibly overcome the constraints and health emergencies imposed on society by the COVID-19 pandemic [57,58]. Drones have been shown to prevent the spread of coronavirus infection by limiting person-to-person contact and stopping the unwanted movement of people during the lockdown [58]. Their independence on road infrastructure and remote operations enables them to be a viable option for various supply chains in hard-to-reach settings [56]. The use of drone technology has been proven to be effective in the delivery of much-needed medical supplies to Rwanda, Lesotho and Ghana’s rural hospitals [59,60,61]. Drone technology has also been shown to be effective in inventory management more efficiently than humans through the movement of items, locating specific inventory, surveying large areas and inspecting labels while saving time and eliminating human error [56]. In addition to diagnostic supplies to remote and hard-to-reach settings, the concept of using inventory drones can be adapted and implemented to help improve the efficiency in monitoring stock levels of POC diagnostic tests at storage facilities. As with other health technologies, the effective and sustainable implementation of this technology will need to be context-specific and include the involvement of all key stakeholders.

### 4.1. Implications for Practice

All the primary studies included in this scoping review were conducted in resource-limited settings in LMICs, as these settings have been reported to have weak health systems and limited coverage or access to diagnostic services. Resource-limited settings have serious SCM barriers. Stockouts of POC tests are a major challenge, and most PHC facilities do not procure POC tests on their own but rely on making requisitions at the district or national level. Distribution to PHC facilities is normally delayed, negatively affecting health outcomes, because patients cannot be tested in a timely manner. A sustainable, well-managed SCM system is important in resource-limited settings, and digital technology may be a viable option. The following SCM measures can be implemented to ensure sustainability: strengthen procurement systems, appropriate forecasting, the efficient training of SCM and health personnel, equitable and timely distribution, efficient inventory management and adequate quality assurance.

### 4.2. Implications for Research

We found limited published research on the SCM of POC diagnostic services in resource-limited settings. Our scoping review revealed that there are currently no primary studies on the SCM systems of COVID-19 POC diagnostic services. We therefore recommend further research to investigate the COVID-19 SCM of POC diagnostic services, explore SCM systems utilised in high-income countries with the aim of adopting sustainable solutions for LMICs and systematic analyses of the impacts of the COVID-19 pandemic on SCM systems. We also recommend primary studies investigating the barriers and enablers of SCM systems in resource-limited settings to provide sustainable solutions to SCM challenges.

### 4.3. Strengths and Limitations

This scoping review is one of the few to comprehensively map the evidence of SCM systems for POC diagnostics services globally with the aim of optimising COVID-19 POC diagnostic services. This scoping review revealed significant gaps in the literature on SCM systems of COVID-19 POC diagnostic services. The use of a scoping review to map evidence allowed the incorporation of different study designs, and we used a transparent and reproducible method to identify, chart, analyse and appraise the articles [15]. The strength of this scoping review is that a comprehensive literature search in relevant electronic databases was conducted. The search included all articles published from inception to date, and no language restrictions were applied. In order to be as comprehensive as possible, the scoping review utilised many keywords and used Medical Subject Heading terms. Despite attempts to be as comprehensive as possible, other published and grey literature may have been missed during the literature search, because COVID-19 is a novel virus, and more research is being published frequently.

This scoping review mapped evidence on the SCM of POC diagnostic services while optimising the COVID-19 testing capacity in resource-limited settings; however, there were no primary studies retrieved that assessed the SCM of COVID-19 POC diagnostic services. It is recommended that primary studies to investigate the SCM of COVID-19 diagnostic services in resource-limited settings are conducted.

## 5. Conclusions

This scoping review revealed that there is limited research on the SCM of POC diagnostic testing in resource-limited settings. POC diagnostic services are fundamental in the timely control and management of COVID-19. The supply chain of POC diagnostic testing should be optimised to ensure access to all patients. The adoption of digital technology can play a crucial role in mitigating the SCM challenges arising from the COVID-19 pandemic. There is currently no research focusing on the SCM of POC tests in the control and management of COVID-19. Therefore, there is an urgent need to conduct primary studies that will investigate the SCM for SARS-CoV-2 POC testing services in order to reveal the research gaps and provide evidence-based solutions for policymakers and implementers of this service.

## Figures and Tables

**Table 1:** PCC framework for determining the eligibility of the research question.

**Population**	Point of Care (POC) diagnostic services: Diagnostic services that use innovative medical technologies that enable near-patient disease diagnosis [10].
**Concept**	Supply Chain Management (SCM) systems: Resources and processes needed to deliver goods and services to consumers with complete satisfaction in a cost-optimized manner [13,18].
**Context**	Globally

**Table 2:** Characteristics and findings of the studies included in the scoping review.

Author and Year of Publication	Title of Study	Aim of Study	Country	Study Design	Study Setting	Study Population	Type of POC Test Investigated	Stage of SCM Investigated	Main Findings
Kuupiel et al., 2017 [10]	Improving the Accessibility and Efficiency of Point-of-Care Diagnostics Services in Low and Middle-Income Countries: Lean and Agile Supply Chain Management	The review provides an overview of the impact of POC diagnostics on healthcare outcomes and factors that contribute to the accessibility of POC diagnostics in LMICs	Global	Review	Low and middle income	General population	HIV, Syphilis and Malaria rapid diagnostic test	Accessibility and availability of POC tests, Test production, selection, quantification, procurement, storage, distribution, quality assurance, inventory management	Barriers to supply chain: Irregular supply, poor forecasting, selection of appropriate diagnostics, unclear procurement systems, delay distribution systems, poor maintenance of quality assurance, and inadequate stock affect existing diagnostics
Kuupiel et al., 2019 [12]	Supply chain management and accessibility to point-of-care testing in resource-limited settings: a systematic scoping review	The study aimed to map evidence on SCM of and accessibility to POC testing focusing on availability and use of POC tests in LMICs.	Global	Review	Low and middle income	General population	Malaria, Syphilis, HIV, Diabetes, Dyslipidaemia, RST and Hepatitis B virus rapid diagnostic tests	Availability of tests, Stockouts, Quantification, Forecasting, Inventory management, Distribution and Storage	Challenges reported: weak procurement, inventory and stock management, and human resource capacity for SCM resulted in test stockouts as well as, declined use of POC tests. Availability of adequate quality POC diagnostic tests increases access to POC testing and improved healthcare. Need to strengthen quantification and forecasting, procurement, inventory management, distribution systems, quality management systems, and human resource capacity to prevent test stockouts, sustain POC testing services, and maximize the benefits of implementing POC testing programmes in LMICs.
Kuupiel et al., 2019 [21]	Empirical Framework for Point-of-Care Diagnostics Supply Chain Management for Accessibility and Sustainability of Diagnostic Services in Ghana’s Primary Health Care Clinics	The aim of the review is to describe the significance of supply chain management in relation to POC diagnostic tests in rural PHC clinics.	Ghana	Review	Low income	General population	Not specified	Stockouts, Distribution, Storage, Inventory management	SCM challenges reported: poor inventory management, clinic managers mostly do not have the autonomy to purchase medical consumables or supplies such as POC tests on their own, clinic managers mostly request POC tests either from centralised regional/provincial medical stores or from their respective district health directorates, and the timely supply of tests is mostly dependent on availability of the test. Even when the POC tests are made available at various medical stores, supply chain management challenges arise: storage, transportation, quality management, inventory management challenges, and human resource capacity for POC testing may be weak and could threaten the sustainability of the service at the PHC level.
Kuupiel et al., 2019 [22]	Poor supply chain management and stockouts of point-of-care diagnostic tests in Upper East Region’s primary healthcare clinics, Ghana	The aim of the study is to audit the supply chain management for POC diagnostic tests in rural Upper East Region’s (UER) PHC clinics, Ghana to determine the reasons for POC tests deficiencies	Ghana	Review	Low income	General population	Haemoglobin, HIV, Syphilis, Hepatitis B, Blood glucose, Malaria, Urine pregnancy and Urine protein	Inventory management, Selection, Distribution, Stock levels	Inventory management: responsibility of the clinic supervisor/manager within the clinic. Test selection: responsibility of higher authorities at the District, Regional, and National levels (ASSURED guidelines). Distribution: responsibility of the health authorities at the Regional medical store and District Health Directorate upon request by the PHC clinics. Stockouts: due to inadequate inventory management and test stockout at the Regional Medical Store/District Health Directorates
Peeling 2015 [23]	Diagnostics in a digital age: an opportunity to strengthen health systems and improve health outcomes	Re-examine the Achilles heel and explore the promises and challenges of diagnostics in a digital age.	Global	Review	Global coverage	General population	Malaria, HIV and Syphilis rapid diagnostic tests	Access to testing, Quality Assurance, Stockouts	Enablers of SCM: Real-time data monitoring via electronic readers to improve coordination. Data on stocks, device usage and condition can be uploaded via Wi-Fi or cellular networks and transmitted to central databases. By linking the data to SCM software, stockouts can be avoided, health system efficiency improved.
Stevens et al., 2014 [24]	Feasibility of HIV point-of-care tests for resource-limited settings: challenges and solutions	The aim of the study is to outline challenges and solution in the implementation of HIV point of care tests in resource-limited settings	Global	Review	Low and middle income	General population	HIV rapid diagnostic test	Procurement, Production and Quality Assurance	Raises challenges with reimbursement, quality monitoring, lack guideline and regulations
Alemnji et al., 2011 [25]	HIV testing in developing countries: What is required?	This article highlights some of the challenges being faced during decentralization of testing facilities in developing countries and some thoughtful considerations for improving infrastructure and quality systems	Global	Review	Low and middle income	General population	HIV rapid diagnostic test	Procurement, Inventory management	Enablers of SCM: efficient procurement, reagent inventory and stock maintenance, cold chain and establishment of equipment service contracts to ensure uninterrupted, timely and quality testing.
Alemnji et al., 2020 [26]	Building and Sustaining Optimized Diagnostic Networks to Scale-up HIV Viral Load and Early Infant Diagnosis	Reviewing the set of frameworks identified for the effective use of both POC-based and laboratory-based technologies in large-scale VL and EID testing programs among countries, implementing partners, and donors.	Global	Review	Global coverage	General population	HIV rapid diagnostic test	Procurement	The daily challenges that commonly limit the functioning of testing centres: reagent stockouts, inadequate quality assurance and waste management. Moving from laboratory to POC testing does not reduce these challenges, many of which are associated with procurement and supply chain systems. Instead, they may be exacerbated because of the need to manage a larger number of testing sites.
Valera et al., 2021 [27]	COVID-19 Point-of-Care Diagnostics: Present and Future	To analyse the current state of POC technologies for the diagnosis and monitoring of COVID-19 infection and discuss future challenges inCOVID-19 diagnostics	Global	Review	Global coverage	General population	COVID-19 rapid test	Availability of POC Accessibility of POC Procurement Quality assurance	Stakeholders injecting funds to speed up the development of rapid and widely accessible COVID-19 testing. Stakeholders to increase the testing capacity at the POC or at home with new molecular and antigen devices authorized for OTC, at-home testing, the challenges will be to ensure adequate sample collection (to ensure the quality of the test), correct collection technique (to avoid patient harm), and a price that allows continuous access to available tests.
Benda et al., 2021 [28]	COVID-19 Testing and Diagnostics: A Review of Commercialized Technologies for Cost, Convenience and Quality of Tests	Our objective here is to review the commercialized in vitro diagnostic tests for the detection of SARS-CoV-2, primarily focusing on tests granted Emergency Use Authorization (EUA) by the U.S. Food and Drug Administration (FDA).	Global	Review	Global coverage	General population	COVID-19 rapid test	Accessibility and availability of POC Selection	Despite commendable efforts, the pandemic continues to rattle several parts of the world, especially the low- and middle-income households where testing sites are inaccessible and test kits are cost-prohibitive or in limited supply. Conventional test-trace-isolate strategies for SARS-CoV-2 may eventually be replaced by at-home, low-cost, self-testing based on personal preferences. This requires making COVID-19 testing resources easily accessible, affordable, scalable, quicker, and convenient for the general population. At present, it is paramount to ramp up population-scale testing in low and middle-income countries by building a sustainable supply chain logistics.
Poole et al., 2021 [29]	How are rapid diagnostic tests for infectious diseases used in clinical practice: a global survey by the International Society of Antimicrobial Chemotherapy (ISAC)	The study aims to assess the current patterns of use around the world, identify issues for successful implementation and suggest best practice advice on how to introduce new tests.	Global	Review	Global coverage	General population	Influenza, HIV, Hepatitis B, Hepatitis C and Meningitis rapid tests	Availability Quality control	Lower-income countries reported a lower proportion in the availability of rapid tests, but HIV and hepatitis testing were available in greater proportions. HIV and Hepatitis are prevalent in LMICs and are given high priority. The cost of each test is likely to also be a factor in the difference of availability, with multiplexed assays generally being considerably more expensive and requiring more complex logistical support. Methods for reducing the costs of many RDTs are lacking, which limit their availability in low-income settings. There are several existing international regulatory processes for drugs and medications, providing safeguards for their safety and efficacy, they are often lacking for RDTs. As a result, diagnostic tests are often sold and used without any evidence of effectiveness.
Kumar et al., 2021 [30]	Aspects of Point-of-Care Diagnostics for Personalized Health Wellness	The review focuses on practical scenarios associated with miniaturized analytical diagnostic devices at POC application for targeted disease diagnostics smartly and efficiently.	Global	Review	Global coverage	General population	Dengue, TB, HIV, Hepatitis B and COVID-19 rapid tests	Human resource capacity	The major concern for POC testing is to achieve the improvement in accuracy and precision of diagnosis at various stages. To prevent wastages of POC tests appropriate sample handling approaches are required to reduce errors during sampling and measurement.
Fleming et al., 2021 [31]	The Lancet Commission on diagnostics: transforming access to diagnostics	In this Commission, we analyse the current status of diagnostics with the use of the six WHO building blocks of health systems, namely health service delivery, health workforce, health information systems, access to diagnostics (analogous to essential medicines), financing, and leadership and governance, as the basis.	Global	Review	Global coverage	General population	Diabetes, Hypertension, HIV, TB, Hepatitis B, Syphilis, COVID-19 rapid tests	Accessibility Quality assurance	Forty-seven percent of the global population has little to no access to diagnostics. Diagnostics are central and fundamental to quality health care. This notion is under recognised, leading to underfunding and inadequate resources at all levels. The level of primary health care is the diagnostic so-called last mile and particularly affects poor, rural, and marginalised communities globally; appropriate access is essential for equity and social justice. The COVID-19 pandemic has emphasised the crucial role of diagnostics in health care and that without access to diagnostics, delivery of universal health coverage, antimicrobial resistance mitigation, and pandemic preparedness cannot be achieved. Innovations within the past 15 years in many areas (e.g., in financing, technology, and workforce) can reduce the diagnostic gap, improve access, and democratise diagnostics to empower patients. As an example of the potential impact, 1.1 million premature deaths in low-income and middle-income countries could be avoided annually by reducing the diagnostic gap for six priority conditions: diabetes, hypertension, HIV, and tuberculosis in the overall population, and hepatitis B virus infection and syphilis for pregnant women.
Bristow et al., 2015 [32]	A review of recent advances in rapid point-of-care tests for syphilis	The objective of this paper is to assess recent performance data, summarize the latest developments in rapid, point-of care syphilis testing technology and discuss strategies and future directions in the implementation and use of this technology for the prevention and control of syphilis.	Global	Review	Global coverage	Antenatal care clinics	Syphilis rapid diagnostic test	Accessibility, quality assurance	Decentralisation of testing using POC tests can result in increased case finding and treatment for those who need it. The introduction of syphilis rapid tests increased the proportion of antenatal care attendees screened for syphilis to over 90% in all regions that had previously had some testing. WHO helps to make rapid tests available in the places that they are needed through their prequalification program and bulk procurement system. For point-of-care rapid tests to be effective, training on the use and interpretation of tests must be properly provided, and supply chains must be able to sustain access to tests and effective treatment.
WHO 2021 [33]	COVID-19 Supply Chain System Procurement Considerations for COVID-19 Diagnostics	This document aims to bring clarity on the process of requesting and receiving globally sourced COVID-19 critical diagnostics supplies	Global	Website	Low and middle income	General population	COVID-19 rapid test	Procurement Selection Availability of POC	Low- and middle-income countries, as well as some high-income small island developing states, have continued to experience restrictions in test access due to competition for limited volumes with high-income countries. Manufacturers have also faced challenges scaling up manufacturing to meet all testing needs. Prices for diagnostic products remain high and some national governments continue to face restricted access to tests. To access POC tests, purchasers may place orders directly with the companies or utilize one of the available multilateral procurement channels. The funded demand and requests are being followed closely to determine whether these tests may be constrained in volume availability. If they become constrained, an allocation model using the same principles as above will be implemented to ensure equitable distribution.
Betran et al., 2018 [34]	Provision of medical supply kits to improve quality of antenatal care in Mozambique: a stepped-wedge cluster randomised trial	The formative research component of the study assessed factors affecting the implementation of evidence-based antenatal care service	Mozambique	Randomised controlled trial	Low income	Antenatal care clinics	Proteinuria, Anaemia, malaria, HIV, syphilis rapid diagnostic test	Procurement, Distribution, accessibility	Limitations of the health system: packaging of all required supplies and timely delivery of these kits at the clinics addressed weaknesses in the procurement and supply systems. Poor procurement process at higher levels. Scaling up and sustainability were important considerations. Scaling up means assessing the cost effectiveness and ensuring accessibility/availability of the POC test.
Hamer et al., 2012 [35]	Quality and safety of integrated community case management of malaria using rapid diagnostic tests and pneumonia by community health workers	To assess the quality and safety of having community health workers (CHWs) in rural Zambia use rapid diagnostic tests (RDTs) and provide integrated management of malaria and pneumonia.	Zambia	Randomised controlled trial	Low income	Child clinic	Malaria rapid diagnostic test	Inventory management Human resource	Enablers of SCM: transparency provided by well-organized record keeping by CHWs and the engagement of study supervisors to ensure adequate supplies. To strengthen stock management, daily registers and periodic reconciliation of stocks was performed to assess commodity use and ensure that none have passed their expiration dates.
Wahlfeld et al., 2019 [36]	HIV Rapid Diagnostic Test Inventories in Zambézia Province, Mozambique: A Tale of 2 Test Kits	The aim of was to evaluate the inventory levels of HIV RDT kits at healthcare facilities in Zambézia province, Mozambique by identifying patterns of threatened inventory levels and/or stockouts of the RDTs.	Mozambique	Cross-sectional	Low income	General healthcare facilities	HIV rapid diagnostic test	Procurement, Storage, Distribution, Inventory management	Disparities in inventory levels were reported at 3 districts. Barriers to supply chain: insufficient access to, and communication with, the provincial warehouse to be able to avoid the high levels of stockouts that were reported.
Ekambaram et al., 2019 [37]	Analysis of Failure Modes: Case study of Ruggedizing a low-cost Screening Technology in Sub-Saharan Africa	The article outlines the steps taken by the Ukweli Test Strips venture to ensure the quality of the UTI and preeclampsia urinalysis screening strips in Sierra Leone.	Sierra Leone	Case study	Low income	General health facilities	UTI and Preeclampsia urinalysis screening strips	Quality assurance	Potential quality control problems throughout the supply chain (1) original equipment manufacturer: manufacturing standards (2) Boat transport: humidity and temperature (3) Warehouse storage: human error and storage issues (4) Truck transport: humidity, temperature and travel issues (5) District storage: human error and storage issues (6) Motorcycle transport: humidity, temperature and storage issues (7) Clinics and community health care workers: humidity, light, temperature, human error and storage issues
Albertini et al., 2012 [38]	Malaria rapid diagnostic test transport and storage conditions in Burkina Faso, Senegal, Ethiopia and the Philippines	This study aimed to gather data on actual temperatures and humidity levels, in different climatic zones, to which RDTs are subjected as they move through the supply chains that typically serve malaria-endemic countries.	Burkina Faso, Senegal, Philippines and Ethiopia	Cohort study	Low and middle income	General population	Malaria rapid diagnostic test	Storage, Distribution	Storage conditions: high temperatures were recorded at central storage facilities in some countries, conditions were inappropriate for many of the RDTs on the market and frequently exceeded common pharmaceutical storage standards.
Mabey et al., 2012 [39]	Point-of-Care Tests to Strengthen Health Systems and Save Newborn Lives: The Case of Syphilis	Our goal was to determine the feasibility of introducing POCTs into different settings in countries with different health systems and cultural and socio-economic contexts.	Brazil, Peru, Tanzania, Uganda, China and Zambia	Mixed methods	Low and middle income	Antenatal care clinics	Syphilis rapid diagnostic test	Quality assurance, availability of POC	For quality assurance, supervisors were provided with proficiency panels prepared by a reference health care facility. Syphilis POCTs were provided to health facilities through the normal supply chain (training in stock management, record keeping, and quality control) to allow the PIs to monitor supply chain problems and provide sustainable solutions in case of stockouts.
Palmer et al., 2020 [40]	Improving the effectiveness of point of care tests for malaria and anaemia: a qualitative study across three Ghanaian antenatal clinics	This study utilises qualitative interviews to identify the current practice of POCT use, to explore the enablers and barriers to effective implementation of POCT, and to determine how relationships between each of the stakeholder groups may impact on POCT use.	Ghana	Interviews and Focus Group Discussions	Low income	Pregnant women	Malaria and Anaemia rapid diagnostic test	Stockouts, Human resource capacity	Barriers of SCM: lack of consistency in the supply of both mRDTs and HCS to the healthcare facilities, regular stockouts of mRDTs, HCS was unavailable in all three facilities (they were available and consistently supplied only during the trial period), concerns about procurement and regular supply of HCS kits (after the trial period). Health workers reported often having to resort to purchasing mRDTs privately.
Magesa et al., 2020 [41]	Factors associated with stock out of malaria test kit in Oshana region, Namibia	This study focuses its attention on factors associates with stockout of mRDT	Namibia	Mixed methods	Middle income	General health facilities	Malaria rapid diagnostic test	Stockouts, Distribution, Storage	Four themes arose from the study. Theme 1: Stock out of mRDT. Theme 2: Medicine policy and decision makers Theme 3: Shortage of knowledge/training in supply chain logistics. Theme 4: Delays in transportation.
Maddox et al., 2017 [42]	Assessing stakeholder perceptions of the acceptability and feasibility of national scale-up for a dual HIV/syphilis rapid diagnostic test in Malawi	This evaluation explores stakeholder perceptions of a novel, dual HIV/syphilis rapid diagnostic test and potential barriers to national scale-up of the dual test in Malawi.	Malawi	Interviews	Low income	Stakeholders involved in provision of antenatal care	HIV and Syphilis rapid diagnostic test	Stockouts, Procurement	Perceived reasons for HIV and syphilis test kit stockouts: low baseline supply of tests given limited funding, expired test kits or staff unwillingness to conduct the tests because they have not received training. Syphilis test kits were stocked out because they were expired, and people wanted to be trained to use the test kits
Hussain et al., 2013 [43]	Public health system readiness to treat malaria in Odisha State of India	The study attempted to evaluate the system readiness to deploy RDTs and ACT for malaria control across the State through health facility surveys and interviews with community workers.	India	Mixed methods	Middle income	General healthcare facilities and stakeholders in malaria control	Malaria rapid diagnostic test	Stockouts	Readiness was assessed in terms of the availability of trained human resources, drugs and diagnostics. Despite a high level of knowledge about how best to diagnose and treat malaria, the ability of the peripheral health workers to optimize fever management and malaria diagnosis was compromised by a failure of the supply chain (poor availability of POC tests due to poor communication/procurement system)
Hasselback et al., 2014 [44]	Rapid diagnostic test supply chain and consumption study in Cabo Delgado, Mozambique: estimating stock shortages and identifying drivers of stockouts	The aim of the study is to estimate malaria RDT stock shortages and the percentage of overall need met by the existing stock in the Cabo Delgado province of Mozambique	Mozambique	Mixed methods	Low income	General health facilities	Malaria rapid diagnostic test	Procurement, stockouts	SCM challenges: procurement for tests was donor supported and requisition-based supply chain has been associated with supply dysfunction (stockouts followed by periods of excessive stock). Supply chains need to respond to timely consumption data to ensure that inventory is appropriately stocked with respect to demand. Stockouts: poor data control and consumption tracking, system responded to an underestimate of the true demand thereby positioning lower inventory than needed in the supply chain.
Dassah et al., 2018 [45]	Rollout of rapid point of care tests for antenatal syphilis screening in Ghana: healthcare provider perspectives and experiences	The aim of the study is to present the perspective of healthcare providers in public health facilities in selected regions of Ghana in relation to their experiences and challenges following a national rollout of rapid syphilis POCTs in Ghana.	Ghana	Mixed methods	Low income	Antenatal care clinics	Syphilis rapid diagnostic test	Stockouts	Interruptions in the supply of syphilis POCTs and penicillin: lack of clear communication channels and poor monitoring and supervision adversely affected implementation of the programme, expired tests kits and failure to replenish stocks, healthcare providers and programme coordinators blamed each other for stockouts.
Blanas et al., 2013 [47]	Barriers to community case management of malaria in Saraya, Senegal: training, and supply-chains	The study evaluates communities’ perceptions of a new community case management of malaria programme in Senegal	Senegal	Mixed methods	Low income	General population	Malaria rapid diagnostic test	Availability, Stockouts, human resource capacity	Stockouts were reported: attributed to inaccurate record-keeping and ignored supply requests, procurement system failures and inadequate central stores.
Boadu et al., 2016 [48]	Challenges with implementing malaria rapid diagnostic tests at primary care facilities in a Ghanaian district: a qualitative study	The aim of the study was to determine which factors influenced RDT implementation for routine malaria management at primary care facilities in the study district.	Ghana	Interviews, focus group discussions, direct observation	Low income	Primary health care facilities	Malaria rapid diagnostic test	Storage, Quality Assurance, Distribution Accessibility	RDT supplies from the district health directorate to their facilities were often insufficient and sporadic. This challenge was more pronounced at remote facilities solely dependent on government supplies and a major hindrance to routine malaria testing at all facilities. At the time of the study four facilities had limited RDTs while two had none. Stockouts were common, sometimes lasting several months, making providers hesitant to use limited quantities when available. Malaria testing at public facilities dependent on government RDT supplies was interrupted due to frequent and prolonged RDT stock outs. Some private providers mentioned purchasing RDTs from independent sources when available. Others abandoned RDT use altogether, citing the economic and technical advantages of microscopy over RDTs.
Asiimwe et al., 2012 [46]	Early experiences on the feasibility, acceptability, and use of malaria rapid diagnostic tests at peripheral health centres in Uganda-insights into some barriers and facilitators	This study and other operational research were conceived and carried out to facilitate evidence-based policy formulation and high-quality implementation of mRDT-led, parasite-based diagnosis.	Uganda	Qualitative Cross-sectional	Low income	General health facilities	Malaria rapid diagnostic test	Selection, Quality Assurance and Safe Disposal	Test selection: Given the predominance of P. falciparum as the cause of malaria in this setting, it was decided to use a histidine rich protein-2 (HRP2) type of mRDT. In deciding the mRDT brand to use, a basic assessment of ease of- use was carried out on four brands amongst nine health workers at a health centre not involved in this study. The brand was chosen on the basis of packaging and labelling, ease of performance, readability of the results, cost, heat stability data, and reported sensitivity and specificity.
Cheng et al., 2016 [49]	Data connectivity: A critical tool for external quality assessment	The aim of the study is to address challenges in training and quality assurance when embedding connectivity in their new POC diagnostic instruments or providing some form of channel for electronic result exchange.	Zimbabwe	Review	Low income	General health facilities	HIV rapid diagnostic test	Quality assurance, Stockouts	Connectivity has shown that it is possible for Ministries of Health to have up-to-the-hour information on testing and test results across the country. These systems can also be twinned to supply chain management software to monitor supplies at each site, providing an automated system for alerts to avoid stockouts.

## Data Availability

All data supporting the conclusions of this scoping review are available through a detailed reference list. The original datasets were not presented, because this scoping review used existing literature.

## Supplementary Materials

The following are available online at https://www.mdpi.com/article/10.3390/diagnostics11122299/s1: File S1: Scoping Review Protocol. File S2: Electronic database search results. File S3: The screening tool. File S4: Full article screening results and agreement. File S5: Search summary table. File S6: MMAT quality appraisal.

## Abbreviations

SARS-CoV-2	Severe acute respiratory syndrome coronavirus type 2
RT-PCR	Reverse transcription polymerase chain reaction
COVID-19	Coronavirus 19
WHO	World Health Organisation
POC	Point of care
SCM	Supply chain management
PRISMA-ScR	Preferred Reporting Items for Systematic Reviews and Meta-Analyses extension for scoping review
MMAT	Mixed method appraisal tool
