# Supplementary material for: A Scoping Review of Supply Chain Management Systems for Point of Care Diagnostic Services: Optimising COVID-19 Testing Capacity in Resource-Limited Settings
Source: Diagnostics (Basel). 2021 Dec 8;11(12):2299. doi: 10.3390/diagnostics11122299 (PMC8700402; doi:10.3390/diagnostics11122299)
Supplement: Supplementary file 1 [file diagnostics-11-02299-s001.zip › Supplementary Material File S1 Scoping Review Protocol.pdf]

diagnostics

A Scoping Review Protocol for supply chain management systems for point of care diagnostic services: Optimising COVID-19 testing capacity in resource-limited settings

Kuhlula Maluleke[1], Alfred Musekiwa[1], Kabelo Kgarosi[2], Sphamandla Nkambule[3], Thobeka Dlangalala[1], Tivani Mashamba-Thompson[4]

[1]School of Health Systems and Public Health, Faculty of Health Science, University of Pretoria, Pretoria, South Africa
[2]Department of Library Services, Faculty of Health Sciences, University of Pretoria, South Africa
[3]Discipline of Public Health Medicine, School of Nursing and Public Health, University of KwaZulu-Natal, South Africa
[4]Faculty of Health Sciences, University of Pretoria, South Africa

Corresponding author: Kuhlula Maluleke

[1]School of Health Systems and Public Health, Faculty of Health Science, University of Pretoria, Pretoria, South Africa

Email: u15266304@tuks.co.za

Citation: Maluleke, K.; Musekiwa, A.; Kgarosi, K.; Gregor, E.M.; Dlangalala, T.; Nkambule, S.; Mashamba-Thompson, T. A Scoping Review of Supply Chain Management Systems for Point of Care Diagnostic Services: Optimising COVID-19 Testing Capacity in Resource-Limited Settings. ***Diagnostics*** **2021**, *11*, 2299. https://doi.org/10.3390/diagnostics11122299

Academic Editor: Chao-Min Cheng

Received: 15 October 2021
Accepted: 17 November 2021
Published: 24 November 2021

Publisher's Note: MDPI stays neutral with regard to jurisdictional claims in published maps and institutional affiliations.

Copyright: © 2021 by the authors. Licensee MDPI, Basel, Switzerland. This article is an open access article distributed under the terms and conditions of the Creative Commons Attribution (CC BY) license (https://creativecommons.org/licenses/by/4.0/).

Abstract: Background: Globally, point of care (POC) tests are being rapidly developed for SARS-CoV-2. To ensure equitable availability and accessibility of these tests, efficient supply chain management (SCM) systems are essential. Here we outline a protocol for a scoping review aimed at mapping evidence on SCM for POC diagnostic services in resource-limited settings. **Methodology:** The proposed scoping review will be guided by an adapted version of the Arksey and O'Malley methodological framework. We will search for published literature on multiple electronic databases and government websites for studies presenting evidence of SCM for POC diagnostics services. We will include articles reporting evidence published since inception. Language restrictions will not be applied. **Discussion:** The proposed scoping review will map relevant evidence on SCM systems for POC diagnostic services globally. We anticipate finding relevant literature that can be synthesized to help guide both future research and provide evidence to guide implementation of sustainable SCM for SARS-CoV-2 POC diagnostic services in resource-limited settings.

Keywords: Point of care diagnostic services; supply chain management; COVID-19; resource-limited settings.

Background

The World Health Organisation (WHO) recommends using reverse transcription polymerase chain reaction (RT-PCR) tests for detecting severe acute respiratory syndrome coronavirus type 2 (SARS-CoV-2) as a gold standard for diagnosing coronavirus disease-2019 (COVID-19) [1-3]. SARS-CoV-2 RT-PCR tests are available for use at point-of-care (POC) and they have been shown to produce reliable results at primary healthcare level [4]. Despite the high performance and reliability of RT-PCR, the availability and public health impact of these tests in resource-limited settings is constrained by availability of testing capacity, shortages of reagents/supplies, lack of skilled laboratory personnel, and costs [5-7]. In resource-limited settings this has resulted in a large backlog when testing patient samples suspected of SARS-CoV-2 [8]. In this study, we define resource-limited settings as settings characterized with having limited access to laboratory infrastructure and limited capability to provide care for life-threatening illness and limited basic critical care resources. Alternative diagnostic methods such as point of care (POC) testing may ease the burden on healthcare facilities and laboratory services in these settings [1,2]. POC testing refers to diagnostic testing that uses innovative medical technologies that enable near patient disease diagnosis to inform clinical decisions [6]. Acceptable POC tests for resource-limited settings are expected to have the following characteristics: affordability, easy to perform, produce rapid results and can be used at site of triage and outside health care facilities to guide disease management [1,6]. Low cost SARS-CoV-2 tests such as the cepheid xpert xpress SARS-CoV-2 antigen rapid tests are currently for use in resource limited settings [9].

POC tests deliver prompt results, therefore, they are of importance in containing highly infectious diseases such as SARS-CoV-2 [6]. The WHO recommended scaling up testing programmes for SARS-CoV-2 by testing all suspected cases [10]. This recommendation was prompted by a resurgence of COVID-19 and the continuum of limited access to diagnostic services in 47% of the global population [11,12]. The COVID-19 pandemic has emphasised the critical role of diagnostics in health care and that without access to diagnostics, delivery of universal health coverage and pandemic preparedness cannot be achieved because for the efficient control and management of COVID-19, POC tests are required on a large scale [12,13]. Globally, POC tests are being rapidly developed for SARS-CoV-2 [14]. To ensure equitable availability and accessibility of POC tests, efficient supply chain management (SCM) is necessary. Supply chain refers to resources and processes needed to deliver goods and services to consumers with complete satisfaction in a cost-optimized manner [15,16]. Optimal SCM of SARS-CoV-2 POC tests will ensure that health care professionals have the available resources to perform tests for patients [6]. An ineffective SCM may limit the availability and accessibility of POC tests and negatively impact health outcomes [16,17]. Evidence on supply chain systems for POC diagnostics is not clear. The purpose of this study is to map evidence of SCM systems of all existing POC diagnostic services in order to reveal gaps that can guide literature and to present evidence that can add to the body of knowledge as well as guide implementers on implementing sustainable SCM for POC diagnostics in order to help improve SARS CoV-2 testing capacity in resource-limited settings.

Methodology

The proposed scoping review will be conducted as a first phase of a multi-phase PhD study that is aimed at developing a novel approach for improving supply chain management for SARS-CoV-2 point of care diagnostic services in resource-limited settings. The proposed scoping review will be aimed at mapping evidence on SCM systems for POC diagnostic services. We propose to conduct a scoping review with guidance from the Arksey and O'Malley [18] framework and further advanced by Levac et al. [19]. This framework entails following 6 steps namely; identification of the research question, identification of relevant articles, selection of eligible articles, charting the data, collating, summarising and reporting of result, and an optional consultation with key stakeholders. This scoping review will not engage in consultation with key stakeholders.

Identification of the research question

Our research question is: What is the evidence on SCM systems for POC diagnostics services, globally? We used the Population, Concept and Context (PCC) framework to determine the eligibility of our research question for the scoping review (Table 1).

Table S1: Determining the eligibility of the research question using Population, Concept and Context (PCC) framework

**Population**	**Point of Care (POC) diagnostics services**<br><br>Diagnostic services that use innovative medical technologies that enable near patient disease diagnosis to guide clinical decision [6].
**Concept**	**Supply Chain Management (SCM) systems**<br><br>Resources and processes needed to deliver goods and services to consumers with complete satisfaction in a cost-optimized manner [15,16].
**Context**	**Globally**

Identification of relevant articles

A comprehensive literature search will be conducted in the following electronic databases: Medline Ovid, Medline Elton B. Stephens Company (EBSCO), Scopus, PubMed, PsycInfo, Web of Science and EBSCOHost. In addition, we will search for gray literature in the form of dissertations/theses, conference proceedings, websites of international organisations such as WHO and government reports. Reference lists of the included articles will also be searched.

To ensure that all relevant articles are included, the comprehensive search strategy will be developed by the Principal Investigator (PI) with the assistance of a subject specialist and university librarian. The search strategy will include the following keywords: 1) “supply chain management” or “supply chain” or “supply chain flow” or “supply chain systems” 2) “point of care” or “point of care testing” or “point of care diagnosis” or “point of care diagnostic services” 3) “SARS-CoV-2” or “COVID-19” or “Coronavirus”. Boolean terms (AND and OR) will be used to separate the keywords. Medical Subject Heading (MeSH) terms will also be used in the electronic database search. Date and language will not be applied. The keywords and MeSH terms will be refined to suit each electronic database search. The results from each electronic database search will be documented in detail, showing the keywords, Boolean terms, MeSH terms, date of search, name of electronic database and number of retrieved articles. We piloted the search strategy on one of the electronic databases and the results of the search are tabulated in table 2.

Table S2: Results of pilot search in PubMed

Date of search	Electronic Database	Keywords and MeSH terms	Number of retrieved studies
25/10/2021	PubMed	(("point-of-care testing"[MeSH Terms] OR "point-of-care systems"[MeSH Terms] OR "point of care testing" OR "Point-of-Care Systems" OR "Point-of-care Testing" OR "Diagnostic Test" OR "Point Of Care System" OR "Point Of Care" OR POCT) AND ("Supply Chain Management" OR "Supply Chains" OR "Supply Chain" OR Logistics OR "Supply Chain Management (SCM)" OR "Sustainable Supply Chains" OR "SCM" OR "Sustainable Supply Chain Management" OR "Supply Chain Management System" OR "Supply-chain Management" OR "Chain Management" OR "Supply Chain Management Practices" OR "Logistics And Supply Chain Management")) AND (("COVID-19 Testing"[Mesh]) AND ("COVID-19 Serological Testing"[Mesh] OR "COVID-19 Nucleic Acid Testing"[Mesh] OR "COVID-19"[Mesh] OR "SARS-CoV-2"[Mesh]) OR "COVID-19" OR "Coronavirus Disease 2019" OR "SARS-CoV-2" OR "Severe Acute Respiratory Syndrome Coronavirus 2" OR Coronavirus "Covid-19" OR "SARS Coronavirus")	467

We will optimise our search strategy by adopting the search summary table (SST) outlined by Bethel et al [20]. The SST will be used to improve and report on the effectiveness of the search strategy to

ensure the retrieval of high-quality, relevant and scientifically sound articles. The search strategy will continuously be improved. An update search (re-run) is essential because SARS-CoV-2 is a novel virus and new research is published frequently.

Selection of eligible articles

Selection criteria were developed to ensure that relevant articles on SCM for POC diagnostic services will be included.

Inclusion criteria

Articles will be included in the scoping review if they have the following characteristics:

- Reporting evidence on SCM systems of all diseases
- Reporting evidence of SCM systems for all POC diagnostic services at all levels of the healthcare continuum
- Reporting evidence of primary studies conducted in LMICs
- Reviews providing evidence of SCM systems for all POC diagnostic services
- Published since inception

Exclusion criteria

Articles will be excluded from the scoping review if they have the following characteristics:

- Lack evidence on SCM systems for all POC diagnostics services
- Reporting SCM systems of laboratory based POC diagnosis
- Reporting evidence of primary studies conducted in high income countries

Following the database search, articles with relevant titles will be exported to an Endnote 20 library and duplicates will be removed. A screening tool will be created using Google form and piloted prior to screening. Screening will be done in two stages by two independent reviewers. Firstly, we will screen for abstracts. Disagreements at this stage will be resolved through discussions until a consensus is reached. Secondly, we will screen the full texts of the articles that made it past the abstract screening stage. Disagreements at this stage will be resolved by inviting a third reviewer.

To determine the inter-rater level of agreement between the two reviewers, the Cohen's kappa statistic will be calculated. The kappa statistic will be interpreted as follows: < 0.1 will represent no agreement and 0.10-0.20 will represent none to slight agreement, 0.21-0.40 will represent fair agreement, 0.41-0.60 will represent moderate agreement, 0.61-0.80 will represent substantial agreement, and 0.81-1.00 will represent almost perfect agreement. The process of study selection will be reported using the Preferred Reporting Items for Systematic Reviews and Meta-Analyses extension for scoping review (PRISMA-ScR) flow chart as depicted in figure 1, and will be updated once the review process is completed [19].

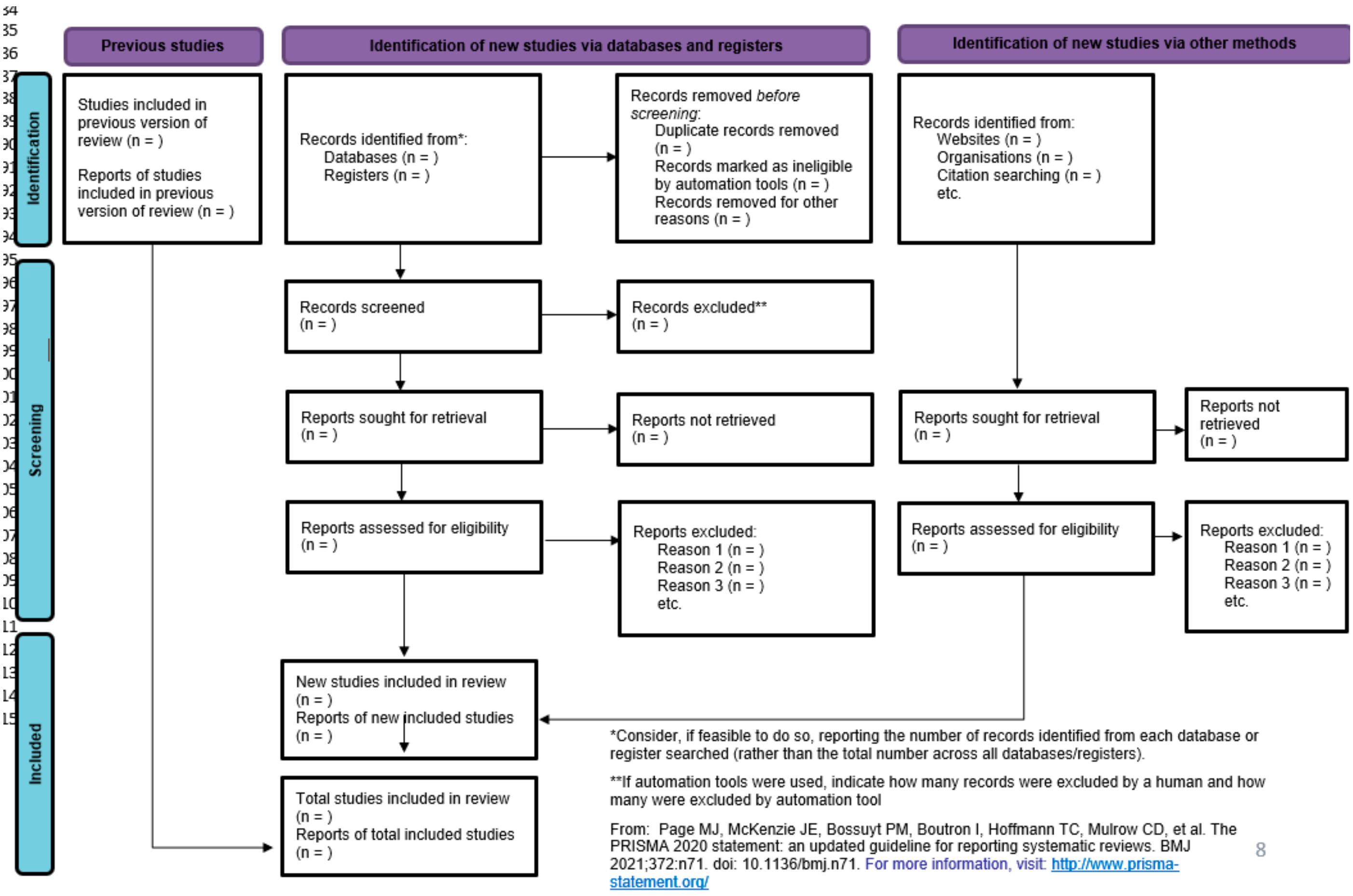

Figure S1: Preferred Reporting Items for Systematic Reviews and Meta-Analyses extension for scoping review (PRISMA-ScR) flow chart

Charting the data

A data charting form was developed to extract relevant data from each eligible article (Table 3). Two independent reviewers will pilot the data charting form using a random sample of 5 included studies for consistency. We will modify the data charting form as required based on feedback from the two reviewers. We will constantly update the data charting form throughout the duration of the scoping review.

Table S3: Data charting form

Author & year of publication
Title of study
Aim of study
Country
Study design
Study setting
Study population
Type of point-of-care test investigated
Stage of SCM investigated

Main findings
Other significant findings

Collating, summarizing and reporting the results

The results will be described in the form of a table and graphs. We will include a map showing the countries where the studies were conducted. Key findings will be presented in themes. A thematic summary will describe how the findings from the included studies relate to the research question.

Quality appraisal

The mixed method appraisal tool (MMAT) version 2018 will be adopted to assess the quality of the included primary studies [21]. The MMAT tool will enable us to appraise a variety of study methods, i.e. qualitative, quantitative or mixed methods studies [21]. In this quality appraisal, we will explore the following domains: aim of the study, clarity of the research question, appropriate methodology, study design, data sources, sampling technique, data collection procedures and participant recruitment. Quality appraisal involves judgement making therefore, two independent reviewers will carry out the quality appraisal process. An overall percentage quality score will be allocated for each included study. The percentage quality score will be graded ranging from ≤50% which will represent low quality evidence; 51-75% which will represent average quality evidence and 76-100% which will represent high quality evidence.

Ethical considerations

This scoping review involves synthesis of current evidence therefore ethical approval is not required.

Discussion

To control the spread of SARS-CoV-2, governing bodies across the globe enforced travel restrictions to limit the movement of people and as a result it negatively impacted the SCM systems globally [22]. The activities of the SCM systems are interconnected hence an interruption in one of the functions leads to a ripple effect encompassing other functions [23]. The lockdowns enforced by governments restricted the movement of vehicles therefore preventing timely delivery of products to the consumers. Manufacturers source material from all around the globe therefore the sudden closure of international suppliers caused supply disruption for manufacturers [23]. The WHO has encouraged all countries to scale up SARS-CoV-2 testing services leading to an increase in demand for SARS-CoV-2 POC tests. The production capacity was disrupted due to reduced working hours and employees having to alternate working hours in order to maintain social distancing. The SCM system could not respond effectively to the demand resulting in shortages of POC tests [24]. The pandemic caused a shift from physical operations to fully online operations. Many entities are struggling to implement logistical solutions to meet the new demand [23]. The limited social interaction among supply chain partners makes it harder for them to implement collaborative approaches.

The SCM disruptions affected mostly resource-limited setting therefore policymakers have been working around the clock to ensure that supplies of essential medical equipment remain uninterrupted in these settings [22]. To optimise the supply of essential medical equipment, efficient SCM

operations are needed to ensure accessibility and availability of POC tests, especially since the WHO has encouraged all countries to scale up SARS-CoV-2 testing services. To ensure that all countries have enough supplies of SARS-CoV-2 POC tests, the WHO established the COVID-19 supply chain system (CSCS) that provides essential SARS-CoV-2 supplies to all countries [25]. The CSCS coordinates multiple-channel procurement and distribution through the identification of demand, demand aggregation, forecasting, certification, market scanning, sourcing, allocation and delivery of essential supplies to where they are needed most at national and subnational level [25].

The proposed scoping review will collate global evidence on SCM systems of POC diagnostics services published since inception. The scoping review will include all literature published presenting evidence on SCM and POC diagnostics published in any language. Despite attempts to be as comprehensive as possible, other published and gray literature may be missed during the literature search because COVID-19 is a novel virus and new research is published frequently. Managing SARS-CoV-2 requires accurate laboratory diagnosis and POC testing can supplement laboratory testing to boost testing capacity in resource-limited settings with poor laboratory infrastructure. In this scoping review, we will exclude all articles reporting SCM systems of laboratory diagnosis and focus solely on POC testing to provide a clear overview of the available research evidence.

We anticipate that the results of the proposed scoping review will provide a comprehensive insight on the evidence of SCM systems of POC diagnostics services globally and reveal research gaps. Our review will also guide implementation of SARS-CoV-2 POC diagnostics in resource-limited settings. The results will be published in a scientific journal, presented at relevant conferences and form part of workshops with key stakeholders involved in SCM systems for POC diagnostic services.

Availability of data and materials
All data generated or analysed during this study will be included in the scoping review article.

Abbreviations
SARS-CoV-2: Severe acute respiratory syndrome coronavirus type 2
RT-PCR: Reverse transcription polymerase chain reaction
COVID-19: Coronavirus 19
WHO: World Health Organisation
POC: Point of care
SCM: Supply chain management
PRISMA-ScR: Preferred Reporting Items for Systematic Reviews and Meta-Analyses extension for scoping review
MMAT: Mixed method appraisal tool

Acknowledgements
The authors would like to extend their appreciation to Ninety-One SA (Pty) Ltd for paying tuition fees and providing a stipend for the PI, Dr. Cheryl Tosh for editing and the University of Pretoria Faculty of Health Sciences Library Services for their assistance with optimising the search strategy.

Funding
This publication has been made possible by the funding from UNICEF and with support from Future Africa, the University of Pretoria.

Author Information
Affiliation
School of Health Systems and Public Health, Faculty of Health Science, University of Pretoria, Pretoria, South Africa. Kuhlula Maluleke, Alfred Musekiwa, Thobeka Dlangalala
Department of Library Services, Faculty of Health Sciences, University of Pretoria, South Africa. Kabelo Kgarosi
Discipline of Public Health Medicine, School of Nursing and Public Health, University of KwaZulu-Natal, South Africa. Sphamandla Nkambule
Faculty of Health Sciences, University of Pretoria, South Africa. Tivani Mashamba-Thompson
Contributions
KM conceptualised and wrote the draft protocol under the supervision of TMT. SM contributed to the development of the methodology. KB optimised the search strategy. KM prepared the draft manuscript, TMT, AM and TD critically reviewed it. All the authors contributed to the reviewed draft version of the manuscript and approved the final version.
Corresponding author
Correspondence to Kuhlula Maluleke
Ethics declarations
Ethics approval and consent to participate
Ethics approval is not applicable for this scoping review protocol.
Consent for publication
Not applicable.
Competing interests
The authors declare that they have no competing interests.

References
1. Ricco, M.; Ferraro, P.; Gualerzi, G.; Ranzieri, S.; Henry, B.M.; Said, Y.B.; Pyatigorskaya, N.V.; Nevolina, E.; Wu, J.; Bragazzi, N.L.; et al. Point-of-Care Diagnostic Tests for Detecting SARS-CoV-2 Antibodies: A Systematic Review and Meta-Analysis of Real-World Data. *J Clin Med* **2020**, *9*, doi:10.3390/jcm9051515.
2. Peeling, R.W.; Mabey, D. Point-of-care tests for diagnosing infections in the developing world. *Clin Microbiol Infect* **2010**, *16*, 1062-1069, doi:10.1111/j.1469-0691.2010.03279.x.
3. Tahamtan, A.; Ardebili, A. Real-time RT-PCR in COVID-19 detection: issues affecting the results. *Expert Rev Mol Diagn* **2020**, *20*, 453-454, doi:10.1080/14737159.2020.1757437.
4. Leber, W.; Lammel, O.; Monika, R.-F.; Panovska-Griffiths, J.; Czypionka, T. Comparing the Diagnostic Accuracy of Point-of-Care Lateral Flow Antigen Testing for SARS-CoV-2 with RT-PCR in Primary Care (REAP-2). **2021**, doi:10.2139/ssrn.3796103.

5. Cheng, M.P.; Papenburg, J.; Desjardins, M.; Kanjilal, S.; Quach, C.; Libman, M.; Dittrich, S.; Yansouni, C.P. Diagnostic Testing for Severe Acute Respiratory Syndrome-Related Coronavirus 2: A Narrative Review. *Ann Intern Med* **2020**, *172*, 726-734, doi:10.7326/M20-1301.
6. Kuupiel, D.; Bawontuo, V.; Mashamba-Thompson, T.P. Improving the Accessibility and Efficiency of Point-of-Care Diagnostics Services in Low- and Middle-Income Countries: Lean and Agile Supply Chain Management. *Diagnostics (Basel)* **2017**, *7*, doi:10.3390/diagnostics7040058.
7. Girdwood, S.; Carmona, S.; Hannay, E.; Nichols, B. Cost-effectiveness of sars-cov-2 rapid antigen testing in lowresource settings. *Topics in Antiviral Medicine* **2021**, 269-269.
8. Arumugam, A.; Faron, M.L.; Yu, P.; Markham, C.; Wu, M.; Wong, S. A Rapid SARS-CoV-2 RT-PCR Assay for Low Resource Settings. *Diagnostics (Basel)* **2020**, *10*, doi:10.3390/diagnostics10100739.
9. Mboowa, G. Current and emerging diagnostic tests available for the novel COVID-19 global pandemic [version 1; peer review: 2 approved]. *AAS Open Research* **2020**, *3*, doi:10.12688/aasopenres.13059.1.
10. National Institute for Communicable Diseases. The use of antigen testing for diagnosis of SARS-CoV-2 in South Africa. Available online: https://www.nicd.ac.za/wp-content/uploads/2020/12/COVID-19-Antigen-Testing-Guidelines.pdf (accessed on 18/05/2021).
11. Lone, S.A.; Ahmad, A. COVID-19 pandemic - an African perspective. *Emerg Microbes Infect* **2020**, *9*, 1300-1308, doi:10.1080/22221751.2020.1775132.
12. Fleming, K.A.; Horton, S.; Wilson, M.L.; Atun, R.; Destigter, K.; Flanigan, J.; Sayed, S.; Adam, P.; Aguilar, B.; Andronikou, S.; et al. The Lancet Commission on diagnostics: transforming access to diagnostics. *The Lancet* **2021**, doi:10.1016/s0140-6736(21)00673-5.
13. Peeling, R.W.; Wedderburn, C.J.; Garcia, P.J.; Boeras, D.; Fongwen, N.; Nkengasong, J.; Sall, A.; Tanuri, A.; Heymann, D.L. Serology testing in the COVID-19 pandemic response. *The Lancet Infectious Diseases* **2020**, *20*, e245-e249, doi:10.1016/s1473-3099(20)30517-x.
14. Ravi, N.; Cortade, D.L.; Ng, E.; Wang, S.X. Diagnostics for SARS-CoV-2 detection: A comprehensive review of the FDA-EUA COVID-19 testing landscape. *Biosens Bioelectron* **2020**, *165*, 112454, doi:10.1016/j.bios.2020.112454.
15. Pinna, R.; Carrus, P.P.; Marras, F. Emerging Trends in Healthcare Supply Chain Management — An Italian Experience. In *Applications of Contemporary Management Approaches in Supply Chains*; 2015.
16. Francis, J.R. COVID-19: Implications for Supply Chain Management. *Front Health Serv Manage* **2020**, *37*, 33-38, doi:10.1097/HAP.0000000000000092.
17. Kuupiel, D.; Bawontuo, V.; Drain, P.K.; Gwala, N.; Mashamba-Thompson, T.P. Supply chain management and accessibility to point-of-care testing in resource-limited settings: a systematic scoping review. *BMC Health Serv Res* **2019**, *19*, 519, doi:10.1186/s12913-019-4351-3.
18. Arksey, H.; O'Malley, L. Scoping studies: towards a methodological framework. *International Journal of Social Research Methodology* **2005**, *8*, 19-32, doi:10.1080/1364557032000119616.
19. McGowan, J.; Straus, S.; Moher, D.; Langlois, E.V.; O'Brien, K.K.; Horsley, T.; Aldcroft, A.; Zarin, W.; Garitty, C.M.; Hempel, S.; et al. Reporting scoping reviews-PRISMA ScR extension. *J Clin Epidemiol* **2020**, *123*, 177-179, doi:10.1016/j.jclinepi.2020.03.016.
20. Bethel, A.C.; Rogers, M.; Abbott, R. Use of a search summary table to improve systematic review search methods, results, and efficiency. *J Med Libr Assoc* **2021**, *109*, 97-106, doi:10.5195/jmla.2021.809.

21. Hong, Q.N.; Fàbregues, S.; Bartlett, G.; Boardman, F.; Cargo, M.; Dagenais, P.; Gagnon, M.-P.; Griffiths, F.; Nicolau, B.; O'Cathain, A.; et al. The Mixed Methods Appraisal Tool (MMAT) version 2018 for information professionals and researchers. *Education for Information* **2018**, *34*, 285-291, doi:10.3233/efi-180221.
22. Illahi, U.; Mir, M.S. Maintaining efficient logistics and supply chain management operations during and after coronavirus (COVID-19) pandemic: learning from the past experiences. *Environ Dev Sustain* **2021**, 1-22, doi:10.1007/s10668-020-01115-z.
23. Chowdhury, P.; Paul, S.K.; Kaisar, S.; Moktadir, M.A. COVID-19 pandemic related supply chain studies: A systematic review. *Transp Res E Logist Transp Rev* **2021**, *148*, 102271, doi:10.1016/j.tre.2021.102271.
24. Queiroz, M.M.; Ivanov, D.; Dolgui, A.; Fosso Wamba, S. Impacts of epidemic outbreaks on supply chains: mapping a research agenda amid the COVID-19 pandemic through a structured literature review. *Ann Oper Res* **2020**, 1-38, doi:10.1007/s10479-020-03685-7.
25. WHO. COVID-19 Supply Chain System: Requesting and receiving supplies. Available online: (accessed on 18 May).
