# Supplementary material for: A Scoping Review of Supply Chain Management Systems for Point of Care Diagnostic Services: Optimising COVID-19 Testing Capacity in Resource-Limited Settings
Source: Diagnostics (Basel). 2021 Dec 8;11(12):2299. doi: 10.3390/diagnostics11122299 (PMC8700402; doi:10.3390/diagnostics11122299)
Supplement: Supplementary file 1 [file diagnostics-11-02299-s001.zip › Supplementary Material File S2 Electronic Database Search Results.pdf]

A Scoping Review for supply chain management systems for point of care diagnostics services: Optimising COVID-19 testing capacity in resource-limited settings.

Kuhlula Maluleke[1], Thobeka Dlangalala[1], Alfred Musekiwa[1], Kabelo Kgarosi[2], Emily Mac Gregor[3], Sphamandla Nkambule[4], Tivani Mashamba-Thompson[5]

Database search results

Date of search	Electronic Database	Keywords/MeSH terms	Number of retrieved studies
07/06/2021	PubMed	("Supply Chain Management" OR "Supply Chains" OR "Supply Chain"OR Logistics OR "Supply Chain Management (SCM)" OR "Sustainable Supply Chains" OR "SCM" OR "Sustainable Supply Chain Management" OR "Supply Chain Management System" OR "Supply-chain Management" OR "Chain Management" OR "Supply Chain Management Practices" OR "Logistics And Supply Chain Management") AND (point of care testing" OR "Point-of-Care Systems" OR "Point-of-care Testing" OR "Diagnostic Test" OR "Point Of Care System" OR "Point Of Care" OR "POCT)	52
09/06/2021	Ebsco Medline	(("supply chain management") OR (""Supply Chain*) OR "SCM" OR ("Sustainable Supply Chains") OR ("Supply Chain Management System") OR ("Supply-chain Management") OR ("Chain Management") OR ("Supply Chain Management Practices") OR ("logistics and supply chain management")) AND ((((MH "Point-of-Care Testing")) OR ("point of care testing") OR ((MH "Point-of-Care Systems")) OR ("Point-of-care Testing") OR ("Diagnostic Test") OR ("Point Of Care System") OR ("Point Of Care*") OR "POCT") AND (("supply chain management") OR (""Supply Chain*) OR "SCM" OR ("Sustainable Supply Chains") OR ("Supply Chain Management System") OR ("Supply-chain Management") OR ("Chain Management") OR ("Supply Chain Management Practices") OR ("logistics and supply chain management")))	81

07/06/2021	Scopus	("Supply Chain Management" OR "Supply Chains" OR "Supply Chain" OR logistics OR "Supply Chain Management (SCM)" OR "Sustainable Supply Chains" OR "SCM" OR "Sustainable Supply Chain Management" OR "Supply Chain Management System" OR "Supply-chain Management" OR "Chain Management" OR "Supply Chain Management Practices" OR "Logistics And Supply Chain Management" "Supply Chain Management" OR "Supply Chains" OR "Supply Chain" OR "Supply Chain Management (SCM)" OR "Sustainable Supply Chains" OR "SCM" OR "Sustainable Supply Chain Management" OR "Supply Chain Management System" OR "Supply-chain Management" OR "Chain Management" OR "Supply Chain Management Practices" OR "Logistics And Supply Chain Management")	187
07/06/2021	EbscoHost (CINAHL)	("point of care testing" OR "Point-of-Care Systems" OR "Point-of-care Testing" OR "Diagnostic Test" OR "Point Of Care System" OR "Point Of Care" OR "POCT") AND ("Supply Chain Management" OR "Supply Chains" OR "Supply Chain" OR "Supply Chain Management (SCM)" OR "Sustainable Supply Chains" OR "SCM" OR "Sustainable Supply Chain Management" OR "Supply Chain Management System" OR "Supply-chain Management" OR "Chain Management" OR "Supply Chain Management Practices" OR "Logistics And Supply Chain Management")	184
10/06/2021	PsychInfo	((Supply Chain) OR (Supply Chain Management) OR SCM) AND ((point "of" care) OR (point "of" care test*) OR (poc OR poct) OR (Point-of-Care System*) OR (Diagnostic Test*))	2
07/06/2021	Ovid Medline	("point-of-care systems" OR "Point-of-care Testing" OR "Point care Testing" OR "Point Care System" OR "Point Care” AND ("Supply Chain Management" OR "Supply Chain manag" OR "Supply Chain" OR "Delivery of Health Care")	288
10/06/2021	Web of Science	("point of care test" OR "Point of Care") AND ("Supply Chain Management" OR "Supply Chain")	46
01/07/2021	ProQuest	("point of care test" OR "Point of Care") AND ("Supply Chain Management" OR "Supply Chain")	28
28/10/2021	PubMed	Search: (("point-of-care testing"[MeSH Terms] OR "point-of-care systems"[MeSH Terms] OR "point of care testing" OR "Point-of-Care Systems" OR "Point-of-care	316

		Testing" OR "Diagnostic Test" OR "Point Of Care System" OR "Point Of Care" OR POCT) AND ("Supply Chain Management" OR "Supply Chains" OR "Supply Chain"OR Logistics OR "Supply Chain Management (SCM)" OR "Sustainable Supply Chains" OR "SCM" OR "Sustainable Supply Chain Management" OR "Supply Chain Management System" OR "Supply-chain Management" OR "Chain Management" OR "Supply Chain Management Practices" OR "Logistics And Supply Chain Management")) AND (("COVID-19 Testing"[Mesh]) AND ("COVID-19 Serological Testing"[Mesh] OR "COVID-19 Nucleic Acid Testing"[Mesh] OR "COVID-19"[Mesh] OR "SARS-CoV-2"[Mesh]) OR "COVID-19" OR "Coronavirus Disease 2019" OR "SARS-CoV-2" OR "Severe Acute Respiratory Syndrome Coronavirus 2" OR Coronavirus "Covid-19" OR "SARS Coronavirus") Filters: in the last 1 year	
28/10/2021	Google	("point of care test" OR "Point of Care") AND ("Supply Chain Management" OR "Supply Chain")	8
