# Supplementary material for: A Scoping Review of Supply Chain Management Systems for Point of Care Diagnostic Services: Optimising COVID-19 Testing Capacity in Resource-Limited Settings
Source: Diagnostics (Basel). 2021 Dec 8;11(12):2299. doi: 10.3390/diagnostics11122299 (PMC8700402; doi:10.3390/diagnostics11122299)
Supplement: Supplementary file 1 [file diagnostics-11-02299-s001.zip › Supplementary Material File S3 Screening tool.pdf]

A Scoping Review for supply chain management systems for point of care diagnostics services: Optimising COVID-19 testing capacity in resource-limited setings.

Stage 3: Full-text screening

* Required

1. Email *

2. Reviewer

Mark only one oval.

- KM
- EM
- TD

3. Author & Date

4. Study Title

5. Does the study provide evidence of point of care diagnostics services?

Mark only one oval.

- Yes
- No

6. Does the study provide evidence of point of care diagnostics services performed near/close to the patient?

Mark only one oval.

- Yes
- No

7. Does the study provide evidence of accessibility of POC diagnostic tests?

Mark only one oval.

- Yes
- No

8. Does the study provide evidence of availability of POC diagnostic tests?

Mark only one oval.

- Yes
- No

9. Does the study provide evidence of supply chain management in general?

Mark only one oval.

- Yes
- No

10. Does the study provide evidence of POC diagnostic test production?

Mark only one oval.

Yes

No

11. Does the study provide evidence of POC diagnostic test selection?

Mark only one oval.

Yes

No

12. Does the study provide evidence of POC diagnostic test quantification?

Mark only one oval.

Yes

No

13. Does the study provide evidence of POC diagnostic test procurement?

Mark only one oval.

- Yes
- No

14. Does the study provide evidence of POC diagnostic test storage?

Mark only one oval.

- Yes
- No

15. Does the study provide evidence of POC diagnostic test distribution?

Mark only one oval.

- Yes
- No

16. Does the study provide evidence of POC diagnostic test redistribution?

Mark only one oval.

- Yes
- No

17. Does the study provide evidence of POC diagnostic test quality assurance?

Mark only one oval.

- Yes
- No

18. Does the study provide evidence of POC diagnostic test inventory management?

Mark only one oval.

- Yes
- No

19. Does the study provide evidence of safe disposal of used POC diagnostic tests?

Mark only one oval.

- Yes
- No

20. Does the study provide evidence of negotiations with suppliers for POC diagnostic test supply?

Mark only one oval.

- Yes
- No

21.

Mark only one oval.

- Option 1

This content is neither created nor endorsed by Google.

Google Forms
