# Supplementary material for: A Scoping Review of Supply Chain Management Systems for Point of Care Diagnostic Services: Optimising COVID-19 Testing Capacity in Resource-Limited Settings
Source: Diagnostics (Basel). 2021 Dec 8;11(12):2299. doi: 10.3390/diagnostics11122299 (PMC8700402; doi:10.3390/diagnostics11122299)
Supplement: Supplementary file 1 [file diagnostics-11-02299-s001.zip › Supplementary Material File S4 Full Article Screening Results and Agreement New.pdf]

Full article screening results

1=Yes, 0=No

**Author & Date**	**Reviewer 1 (EM)**	**Reviewer 2 (KM)**
Albert 2020	0	0
Albertini 2012	1	1
Alemnji 2011	1	1
Alemnji 2020	1	1
Astori 2020	0	0
Hamer 2012	1	1
Asiimwe 2012	1	1
Barlow 2018	0	0
Bazuin 2015	0	0
Betran 2018	1	1
Benda 2021	1	1
Blanas 2013	1	1
Botti-Lodovico 2021	0	0
Boadu 2016	1	1
Bristow 2015	1	1
Brown 2018	0	0
Burbano 2018	0	0
Carlo 2015	0	0
Cheng 2016	1	1
Dalvantes 2019	0	0
Daniel 2012	1	0
Dassah 2018	1	1
Everitt 2021	0	0
Ekambaram 2019	1	1
Futrell 2020	0	0
Fleming 2021	1	1
Fragala 2021	0	0
Graham 2021	0	0
Hasselback 2014	1	1
Hussain 2013	1	1
Jani Ilesh 2013	0	0
Kabir 2021	0	0
Kumar 2021	1	1
Kuupiel, Bawontuo 2017	1	1
Kuupiel, Tlou 2019	1	1
Kuupiel, Bawontuo 2019	1	1
Kuupiel, Donkoh 2019	1	1
Koksaldi 2021	0	0
Lauria 2019	0	0
Loeffelholz 2021	0	0

Maddox 2017	1	1
Magesa 2019	1	1
Mattioli 2021	0	0
Palmer 2020	1	1
Parapudi 2021	0	0
Peeling 2017	0	0
Peeling 2015	1	1
Peeling 2009	0	0
Peter 2017	1	0
Peterson	0	0
Poole 2021	1	1
Rahman 2021	0	0
Renju 2021	0	0
Stevens 2014	1	1
Sunyota 2019	0	0
Toskin 2016	0	0
Mabey 2012	1	1
Valera 2021	1	1
Vogels 2021	0	0
Wahlfield 2019	1	1
Wilson 2020	0	0
WHO 2021	1	1

```
         Expected
Agreement   agreement    Kappa   Std. err.      Z     Prob>Z
-----------------------------------------------------------------
 96.77%     50.00%    0.9355    0.1267     7.38     0.0000


Reviewer 1 |     Reviewer 2 (KM)
      (EM) |          0          1 |      Total
-----------+----------------------+----------
         0 |         31          2 |         33
         1 |          0         29 |         29
-----------+----------------------+----------
     Total |         31         31 |         62
```

```
. mcc Reviewer1EM Reviewer2KM

                 |        Controls        |
Cases            |   Exposed   Unexposed  |      Total
-----------------+------------------------+-----------
         Exposed |        31           2  |         33
       Unexposed |         0          29  |         29
-----------------+------------------------+-----------
           Total |        31          31  |         62

McNemar's chi2(1) =      2.00    Prob > chi2 = 0.1573
Exact McNemar significance probability       = 0.5000

Proportion with factor
        Cases       .5322581
        Controls          .5     [95% conf. interval]
                   ---------     --------------------
        difference  .0322581     -.0278506   .0923667
        ratio       1.064516      .9761481   1.160884
        rel. diff.  .0645161     -.0219646   .1509969

        odds ratio         .      .1878091          .   (exact)
```
