# Supplementary material for: A Scoping Review of Supply Chain Management Systems for Point of Care Diagnostic Services: Optimising COVID-19 Testing Capacity in Resource-Limited Settings
Source: Diagnostics (Basel). 2021 Dec 8;11(12):2299. doi: 10.3390/diagnostics11122299 (PMC8700402; doi:10.3390/diagnostics11122299)
Supplement: Supplementary file 1 [file diagnostics-11-02299-s001.zip › Supplementary Material File S5 Search Summary Table New.pdf]

Project Title: A Scoping Review for supply chain management systems for point of care diagnostics services: Optimising COVID-19 testing capacity in resource-limited settings
Contributor Author: Kuhlula Maluleke, Thobeka Dlangalala, Alfred Musekiwa, Kabelo Kgarosi, Emily Mac Gregor, Sphamandla Nka
Documnet Title: Search Summary Table (SST) template

Project											
**Included references**	**Format**	**Database searches (*date run, date re-run*)**							**Supplementary searches**		
		PubMed	***Medline***	***Scopus***	***ProQuest***	***CINAHL***	***Web of Science***	***PsychInfo***	***wss***	***bcs***	***hs***
Albertini 2012	Jnl	x	x			x	x				
Alemnji 2011	Jnl	x	x			x					
Alemnji 2020	Jnl	x	y			x					
Hamer 2012	Jnl	x	x	x		x					
Asiimwe 2012	Jnl	x	x			x					
Betran 2018	Jnl	x	x			x					
Blanas 2013	Jnl	x	x	x			x				
Boadu 2016	Jnl	x	x			x					
Bristow 2015	Jnl	x	x	x							
Cheng 2016	Jnl	n	x	x							
Dassah 2018	Jnl	x	x	x		x					
Ekambaram 2019	Jnl	x	x	x							
Hasselback 2014	Jnl	x	x			x	x				
Hussain 2013	Jnl	n	x	x							
Kuupiel, Bawontuo 2017	Jnl	x	x	x							
Kuupiel, Tlou 2019	Jnl	x	x	x							
Kuupiel, Bawontuo 2019	Jnl	x	x	x							
Kuupiel, Donkoh 2019	Jnl	x	x	x							
Maddox 2017	Jnl	x	x	x			x				
Magesa 2019	Jnl	x	x								
Palmer 2020	Jnl	x	y	x							
Peeling 2015	Jnl	x	x	x			x				
Stevens 2014	Jnl	n	x	x							
Mabey 2012	Jnl	n	n	n						x	
Wahlfield 2019	Jnl	x	x	x							
Valera 2021	Gray	n							x		
WHO 2021	Gray	n							x		
Benda 2021	Jnl	x									
Poole	Jnl	x									
Kumar	Jnl	x									
Fleming	jnl	x									
No. included refs		28	22	16	0	9	5	0	2	1	0
No. unique refs		0	0	0	0	1	0	0	0	0	0
No. refs screened		18	35	32	0	32	33	0			
Yield		52	369	184	28	184	46	2			
Sensitivity		96.55172414	75.86206897	55.17241379	0	31.03448276	17.24137931	0			
Precision		53.84615385	5.962059621	8.695652174	0	4.891304348	10.86956522	0			
			No. database searches carried out =	7							
			Sum of yields =	1206			Overall sensitivity	93.5483871			
			No. refs screened at Ti&Ab =	147			Overall precision	19.73			
			No. refs screened at FT=	41			NNR	5			
			No. of included refs from searching =	29			NNR FT	1			
			Total no. of included refs =	31			NNS	4			

Codes
x = found from the search
y = in database; found when search strategy re-run
n = not in the database
z = in the database; not found using the search strategy
(red) = databases where searches re-run

Format codes
jnl = journal article
ths = PhD thesis

Other codes
NNR = number needed to read. 1/overall precision
NNR FT = number needed to read at FT to find one included reference

Supplementary
fcs = forwards c
bcs = backwards
hs = hand searc
wss= website se
org= from conta
