# Supplementary material for: A Scoping Review of Supply Chain Management Systems for Point of Care Diagnostic Services: Optimising COVID-19 Testing Capacity in Resource-Limited Settings
Source: Diagnostics (Basel). 2021 Dec 8;11(12):2299. doi: 10.3390/diagnostics11122299 (PMC8700402; doi:10.3390/diagnostics11122299)
Supplement: Supplementary file 1 [file diagnostics-11-02299-s001.zip › Supplementary Material File S6 MMAT Quality Appraisal.pdf]

RefID	First author	Year	Title
1	Wahlfeld	2019	HIV Rapid Diagnostic Test Invent
2	Palmer	2020	Improving the effectiveness of poi
3	Magesa	2020	Factors associated with stock out
4	Maddox	2017	Assessing stakeholder perception
5	Hasselback	2014	Rapid diagnostic test supply chai
6	Dassah	2018	Rollout of rapid point of care tests
7	Betran	2018	Provision of medical supply kits to
8	Blanas	2013	Barriers to community case mana
9	Mabey	2012	Point-of-Care Tests to Strengthen
10	Hamer	2012	Quality and safety of integrated co
11	Hussain	2013	Public health system readiness to
12	Albertini	2012	Malaria rapid diagnostic test trans
13	Boadu	2016	Challenges with implementing ma
14	Ekambaram	2019	Analysis of Failure Modes: Case s
15	Asiimwe	2012	Early experiences on the feasibilit

SCREENING QUESTIONS		1.	
S1. Are there clear research questions?	S2. Do the collected data allow to address the research questions?	1.1. Is the qualitative approach appropriate to answer the research question?	1.2. Are the qualitative data collection methods adequate to address the research question?
Yes	Yes		
Yes	Yes	Yes	Yes
Yes	Yes		
Yes	Yes	Yes	Yes
Yes	Yes		
Yes	Yes		
Yes	Yes		
Yes	Yes		
Yes	Yes		
Yes	Yes		
Yes	Yes		
Yes	Yes		
Yes	Yes	Yes	Yes
Yes	Yes		
Yes	Yes	Yes	Yes

QUALITATIVE STUDIES			
1.3. Are the findings adequately derived from the data?	1.4. Is the interpretation of results sufficiently substantiated by data?	1.5. Is there coherence between qualitative data sources, collection, analysis and interpretation?	2.1. Is randomization appropriately performed?
Yes	Yes	Yes	
Yes	Yes	Yes	
			Yes
			Yes
			Yes
Yes	Yes	Yes	
Yes	No	Yes	

2. RANDOMIZED CONTROLLED TRIALS			
2.2. Are the groups comparable at baseline?	2.3. Are there complete outcome data?	2.4. Are outcome assessors blinded to the intervention provided?	2.5 Did the participants adhere to the assigned intervention?
Yes	Yes	Yes	Yes
Yes	Yes	Yes	Yes
Yes	Yes	No	Yes

3. NON-RANDOMIZED STUDIES			
3.1. Are the participants representative of the target population?	3.2. Are measurements appropriate regarding both the outcome and intervention (or exposure)?	3.3. Are there complete outcome data?	3.4. Are the confounders accounted for in the design and analysis?

	4. QUANTITATIVE DESCRIPTIVE		
3.5. During the study period, is the intervention administered (or exposure occurred) as intended?	4.1. Is the sampling strategy relevant to address the research question?	4.2. Is the sample representative of the target population?	4.3. Are the measurements appropriate?
	Yes	Yes	Yes
	Yes	Yes	No
	No	Yes	Yes

E STUDIES			5. M
4.4. Is the risk of nonresponse bias low?	4.5. Is the statistical analysis appropriate to answer the research question?	5.1. Is there an adequate rationale for using a mixed methods design to address the research question?	5.2. Are the different components of the study effectively integrated to answer the research question?
Yes	Yes		
		Yes	Yes
		Yes	Yes
		Yes	Yes
		Yes	Yes
		Yes	Yes
		Yes	Yes
Yes	Yes		
Yes	Yes		

IXED METHODS STUDIES		
5.3. Are the outputs of the integration of qualitative and quantitative components adequately interpreted?	5.4. Are divergences and inconsistencies between quantitative and qualitative results adequately addressed?	5.5. Do the different components of the study adhere to the quality criteria of each tradition of the methods involved?
Yes	Yes	Yes
Yes	Yes	Yes
Yes	Yes	Yes
Yes	Yes	Yes
Yes	Yes	Yes
Yes	Yes	Yes

COMMENTS

Comments	Score
	100%
	100%
	100%
	100%
	100%
	100%
	100%
	100%
	100%
	100%
	100%
Insuffiecient information provided on how the devices monitoring temperature were loaded in transport system	95%
	100%
Type of sampling strategy used not discussed	90%
Data are of reported responses which could mean that there was some degree of measurement error and social bias	92%
